# Homogenous catalysis of peroxynitrite conversion to nitrate by diaryl selenide: a theoretical investigation of the reaction mechanism

**DOI:** 10.3389/fchem.2024.1486175

**Published:** 2024-12-16

**Authors:** Yuan Xue, Carrie Salmon, Valentin Gogonea

**Affiliations:** ^1^ Department of Chemistry and Biochemistry, The University of Mississippi, University, MS, United States; ^2^ Department of Chemistry and Biochemistry, Oberlin College and Conservatory, Oberlin, OH, United States; ^3^ Department of Physics, Kent State University, Kent, OH, United States; ^4^ Department of Chemistry, Cleveland State University, Cleveland, OH, United States; ^5^ Department of Cardiovascular and Metabolic Sciences, Lerner Research Institute, Cleveland Clinic, Cleveland, OH, United States; ^6^ Center for Microbiome and Human Health, Cleveland Clinic, Cleveland, OH, United States

**Keywords:** diaryl selenide, peroxynitrite, nitrate, nitrite, reaction mechanism, substituent effect, potential energy surface, frontier molecular orbitals

## Abstract

Quenching peroxynitrite (a reactive oxidant species) is a vital process in biological systems and environmental chemistry as it maintains redox balance and mitigates damaging effects in living cells and the environment. In this study, we report a systematic analysis of the mechanism of transforming peroxynitrite into nitrate using diaryl selenide in water. Through quantum mechanical calculations, we investigate the dynamic isomerization of peroxynitrite in a homogeneous catalytic environment. The mapped potential energy surfaces (PESs) generated using various methods in conjunction with different basis sets suggest that the isomerization mechanism includes four major steps: the reaction of peroxynitrite with diaryl selenide via oxygen-bound selenium; selenium oxidation in the presence of an appropriate oxidant; oxygen transfer; and ultimately, the generation of nitrate. The molecular orbital analysis suggests a substituent effect on the aromatic ring of diaryl selenide in this reaction. Changes in both molecular orbitals and electrostatic potential highlight the significance of the electron transfer step in ensuring the progression of this reaction.

## Introduction

Peroxynitrite (ONOO^−^), a potent reactive nitrogen peroxide species, is involved in numerous physiological and pathological processes, including inflammation, oxidative stress, and neurodegenerative disorders (Radi, 2018; Xia et al., 2021; Pacher and Szabo, 2006; Pacher et al., 2007). Diaryl selenides are compounds with aromatic rings tethered to selenium, exhibiting unique chemical properties that drive the catalytic conversion of peroxynitrite to nitrate. Recent studies have shown their remarkable catalytic activity toward peroxynitrite in an electrochemical environment (Kalil et al., 2021), and several other investigations have explored the factors influencing the performance of other catalytic systems in peroxynitrite isomerization (Herold et al., 2001; Herold et al., 2004; Storkey et al., 2015; Lamani et al., 2015). Understanding the electronic structure of diaryl selenides plays a crucial role in understanding their catalytic activity toward peroxynitrite isomerization. Selenium, which possesses a higher electron affinity than carbon and sulfur, can facilitate electron transfer processes (within a molecular framework), allowing a variety of reaction pathways either in solution or enzymatic systems (Lothrop et al., 2014; Maroney and Hondal, 2018; Palla et al., 2023). Furthermore, the redox-active selenium centers in diaryl selenides contribute to the catalytic turnover by undergoing reversible redox reactions (Hoover and Seferos, 2019). Another example of peroxynitrite isomerization to nitrate is carried out using water-soluble Fe(III) porphyrins (Jensen and Riley, 2002), where the reaction is efficiently catalyzed through an oxo-ferryl intermediate, O = Fe(IV) (Porph). Likewise, the interaction of peroxynitrite with several hemoproteins, such as metmyoglobin and hemoglobin (Herold et al., 2002), peroxidases (But et al., 2002), cytochrome P450s (Lin et al., 2007), and even inducible NOS oxygenase (Maréchal et al., 2007), has been reported, although the mechanism of interaction and the net reaction vary depending on the nature of the hemoprotein.

Utilizing diaryl selenides to convert peroxynitrite to nitrate represents a promising approach for mitigating the harmful effects caused by peroxynitrite in cellular matrices. However, although sophisticatedly designed experiments have clearly demonstrated the conversion of peroxynitrite to nitrate, the stepwise mechanism of this conversion in solution remains unknown. Uncovering the mechanism of peroxynitrite conversion to nitrate anion under the effect of diaryl selenides can provide invaluable support for novel catalysis designs. Therefore, utilizing both *ab initio* Hartree–Fock (HF) and density functional theory (DFT) methods in conjunction with a variety of Pople’s split-valence basis sets, we investigated this catalytic process by exploring the potential energy surface (PES) produced through quantum mechanical (QM) calculations. With the stationary points anchored at four levels of theory, we simulated homogenous catalysis with reactants immersed in a dielectric medium with the dielectric constant corresponding to water. Because an electron-transfer step is involved in the overall mechanism, we assumed that a suitable oxidant is present in the medium (e.g., hydrogen peroxide) and simulated the electron transfer step by changing the charge of the system after the first reaction step. This change in the total charge produces a discontinuous PES pertaining to systems with different numbers of electrons. The linkage between the two disjunct PESs was established by factoring in calculations of an electron-acceptor species, specifically, in this case, a hydrogen peroxide molecule (*vide infra*).

## Methods

In order to map the PES for the conversion of peroxynitrite to nitrate by diaryl selenide, all structures presented in this paper were calculated using the Gaussian 16 software package (Frisch et al., 2016). All stationary points reported in this study were fully characterized using the *ab initio* HF method in conjunction with three different Pople’s split-valence basis sets, namely, 6-31G(d,p), 6-31+G(d,p), and 6-311+G(d,p), unless otherwise specified. The geometry optimizations were conducted with a tight convergence threshold, and the gradient for each optimized structure was kept below 5 × 10^−5^ E_h_/a_0_. Transition states (TSs) were characterized using the Berny and quasi-Newton methods (Peng and Schlegel, 1993; Hratchian and Schlegel, 2005), with further intrinsic reaction coordinate (IRC) scans to verify that they connect two energy minima. Frequency calculations were performed at the same levels of theory for each anchored stationary point to examine its vibrational nature. For all post-HF computations, the same procedure (i.e., locating and characterizing the stationary points on the PES) was repeated with the DFT Becke 3-parameter Lee–Yang–Parr (B3LYP) (Becke, 1988; Becke, 1993) functional in conjunction with the 6-311+G(d,p) basis set and the Minnesota high nonlocality functional with double the amount of nonlocal exchange, M06-2X (Zhao and Truhlar, 2008), in conjunction with the 6-31+G(d,p) basis set.

Additionally, considering that the PES can change at different levels of quantum mechanical theory, especially when the stationary point characterizations are not consistent at different levels of theory, constrained geometry optimizations or single-point energy calculations on the proposed geometry were conducted to probe the curvature of the surface. When a constrained geometry was applied, the two reactive atom centers involved in bond cleavage and/or formation, as indicated by the corresponding TSs, were separated and frozen at a certain distance, whereas the rest of the atoms in the proposed structure were allowed to fully relax through geometry optimization. The pathway was then plotted based on the distance between the two frozen atoms (in Ångstrom) with respect to the raw electronic energy to elucidate the progress of the reaction.

Orbital hybridization, partial atomic charges, and molecular orbitals (MOs) were determined for selected structures using the Natural Bond Orbital (NBO) program from the Gaussian 16 package (Besler et al., 1990). The electrostatic potential (ESP) maps were generated for each structure using the Merz–Kollman ESP calculation as implemented in the Gaussian 16 program (Besler et al., 1990). The thermodynamic quantities were calculated for 298 K and 1 atm. Solvent effects, specifically that of water, were included with the self-consistent reaction field model (polarizable continuum model [PCM]) (Mennucci et al., 2002). The GaussView 6 program was used to visualize the three-dimensional structures and plot molecular orbitals and energy level diagrams (Dennington et al., 2016).

## Results and discussion

### The mechanism of peroxynitrite isomerization

Uncovering the detailed mechanism is of utmost importance for elucidating the role of diaryl selenides as catalysts and harnessing their catalytic prowess in various applications (e.g., electrochemical sensing [Kalil et al., 2021]). Understanding the catalytic role of diaryl selenide in transforming the more redox-reactive peroxynitrite to the relatively inert nitrate will enable researchers to harness the unique properties of diaryl selenide for therapeutic interventions and environmental remediation strategies. By studying the mechanistic steps involved, researchers can gain insights into the factors influencing the efficiency and selectivity of this reaction and achieve better control of catalysis based on theoretical analyses.

**FIGURE 1:**
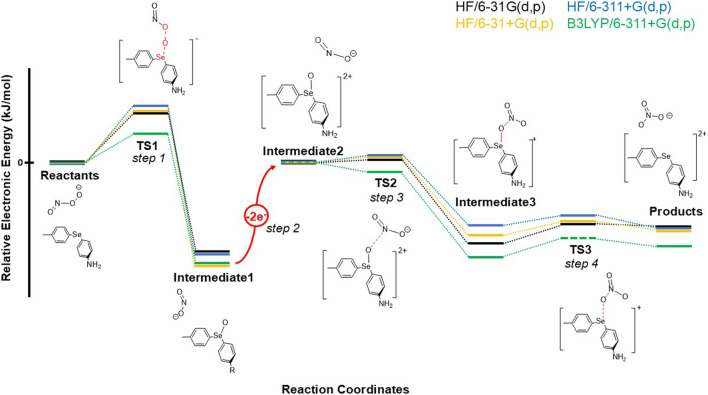
PES diagram at four different levels of QM theory, which depicts a four-step redox reaction mechanism in which peroxynitrite undergoes transformation into nitrate through diaryl selenide catalysis.

To investigate the conversion of peroxynitrite to nitrate catalyzed by one functionalized diaryl selenide, 4-(p-tolylselanyl)aniline, (MePh)Se(PhNH_2_), we determined and anchored all stationary points (optimized geometries) on the PES in the proposed reaction mechanism using QM calculations (both HF and DFT). Our computational results suggested that the conversion of peroxynitrite to nitrate (through homogenous catalysis) with a diaryl selenide catalyst requires four elementary step reactions, as shown in the PES of the reaction in Figure 1. Additionally, based on HF calculations performed as an initial exploration of the mechanism, the results suggest that the triple-ζ 6-311+G(d,p) basis set best balances the computational cost and calculation accuracy.

Although the reaction mechanism mapped through the HF method provides an invaluable insight into the role of diaryl selenide as a catalyst, it is prudent to recalibrate the reaction energetics with a refined electron correlation in order to describe the redox process more accurately. Therefore, the PES and reaction thermodynamics and kinetics were further explored utilizing the DFT method. In order to present helpful information for future relevant investigations on the dynamical oxygen transfer occurring in similar catalyses, our goal was also to calibrate the most affordable *ab initio* method (HF) with trustworthy DFT functionals when describing the electronic structures of various species (reactants, products, transition states, and intermediates) along the reaction pathway for this particular chemical transformation. Numerous DFT functionals have been developed over the years, and their performance in calculating various classes of compounds was thoroughly investigated (Goerigk, L., et al., 2017). We selected the B3LYP and M06-2X functionals to further probe the PES for the isomerization reaction of peroxynitrite. The B3LYP functional was extensively used in the last 30 years, while M06-2X is a more recent functional that performs rather well for non-metallic compounds (Zhao and Truhlar, 2008). However, we believe that the lack of experimental data or high-level *ab initio* results on this system precludes the evaluation of the accuracy of these two functionals with respect to other DFT functionals found in the literature. Additionally, in justifying our choice for a computational protocol, we note that exploratory calculations performed by adding the D3 dispersion correction (Grimme, S., et al., 2011) to the B3LYP functional produce minimal changes in the PES, which suggests that the reaction pathway we reported in this study is not sensitive to further dispersion refinement in computations. Therefore, we utilized the B3LYP and M06-2X functionals without dispersion corrections for all DFT computations (including full geometry optimization and frequency computations; Table 1; Supplementary Tables S2, 4, 5) on the PES for the isomerization reaction, while additional explorations of the post isomerization reaction (e.g., restoring the initial oxidation state of Se and studying the substituent effect) were only based on the electronic structures obtained using the HF level of *ab initio* theory.

**TABLE 1:** Electronic energies (EEs), relative energies (∆E), and activation energies (E_a_) for stationary points anchored on the potential energy surface, as shown in Figure 1.

Step #	Compound	B3LYP/6-311+G(d,p)			HF/6-311+G(d,p)		
		EE^[a]^	∆E^[b]^	E_a_^[c]^	EE^[a]^	∆E^[b]^	E_a_^[c]^
1	Reactants	−3240.138532	0.00	N/A	−3233.258159	0.00	N/A
	TS1	−3240.119327	50.42	50.42	−3233.220138	99.82	99.82
	Intermediate 1	−3240.207611	−181.37	N/A	−3233.317967	−157.03	N/A
2	2e^−^ transfer^[d]^						
3	Intermediate 2^[e]^	−3239.770483	0.00	N/A	−3232.937656	0.00	N/A
	TS2	−3239.776469	−15.72	−15.72	−3232.932308	14.04	14.04
4	Intermediate 3	−3239.833766	−166.15	N/A	−3232.979379	−109.54	N/A
	TS3^[e]^	−3239.821050	−132.76	33.39	−3232.973270	−93.50	16.04
	Products	−3239.826388	−146.78	N/A	−3232.982404	−117.49	N/A

[a] Electronic energy in Hartree.

[b] In kJ/mol, for stationary points anchored in step 1. ∆E (energy relative to intermediate 2) was calculated using the equation ∆E = (E_target molecule_–E_reactants_) × 2,625.5; otherwise, it is determined using equation ∆E = (E_target molecule_–E_intermediate 2_) × 2,625.5.

[c] Activation energy in kJ/mol.

[d] Thermochemistry nature of the surface is listed in Supplementary Table S4 in Supplementary Material. The Cartesian coordinates are listed in Supplementary Table S5.

[e] Structure vanishes when the DFT method is applied due to the nature of the PES (Supplementary Table S1; Supplementary Figure S1). Single-point energetics (in green) at the B3LYP/6-311+G(d,p) level of DFT theory based on structures obtained at the HF/6-311+G(d,p) level of *ab initio* theory.

The dynamical electron correlations cause two notable discrepancies, although the relative energetics probed at the B3LYP/6-311+G(d,p) and M06-2X/6-31+G(d,p) levels of QM theory reinforce the trend observed in the HF calculations. The first special case occurred during step 2 (Figure 1). Based on the fully characterized transition state 2 (TS2), the IRC scan along the vibrational mode would not fully converge to the expected local minimum (intermediate 2) despite the forward scan converging to intermediate 3. We speculate that the inability of the backward scan to anchor intermediate 2 might be due to the robust interactions between the highly polarizable Se core (Steinman et al., 2010; Koppenol, 1999) and the NO_2_^−^ anion, which eliminate the local minimum and make it unresolvable through the DFT calculation at this level of theory. Additionally, the failure of locating intermediate 2 with this computational setup might also be due to the rapid energy change and the discontinuity in the PES at step 2 (two-electron oxidation). By applying constrained geometry optimizations, the PES was carefully explored by scanning along the vibrational mode corresponding to the only imaginary frequency observed in TS2. The DFT scanning results confirm our supposition that a strong O … N interaction occurs almost instantaneously after oxidation as the reaction exhibits exothermicity when the N atom approaches the O atom from 2.91 Å to 2.71 Å with a 0.05-Å step size. This step in the reaction requires a minimal activation energy to overcome TS2 based on the more sophisticated scans near the N-O bond length in TS2 (2.607 Å–2.655 Å with a 0.007-Å step size) (Supplementary Table S1; Supplementary Figure S1). The additional B3LYP/6-311+G(d,p) single-point energy computations on HF/6-311+G(d,p)-optimized structures (further denoted as B3LYP/6-311+G(d,p)//HF/6-311+G(d,p)) also align with the scan results. The calculations indicate that intermediate 3 could be formed immediately through the low-lying TS2. The same scenario could be observed in the remaining steps, suggesting that once the reaction surpasses the rate-determining step 1, the oxidation on the nitrogen center of NO_2_^−^ proceeds rapidly.

From a thermochemistry perspective, our QM calculations at the HF/6-311+G(d,p) level yielded two disjoint segments of the PES. Therefore, prior to delving into the intricacies of the mechanism and molecular orbital analyses, it is crucial to highlight the electron transfer in the second step of the isomerization reaction as it leads to a discontinuity in the PES. The observed discontinuity arises from a change in the total charge of the system, transitioning from −1 to +1 following a two-electron transfer (to a suitable acceptor). The connection between these two segments necessitates an assessment of the energy required for the electron-transfer process. To enable this analysis, we incorporated a suitable oxidant, i.e., hydrogen peroxide (Orian and Flohe, 2021), into the calculations. This allowed for the examination of the electronic structure of both the hydrogen peroxide molecule and its subsequent product post-electron transfer, i.e., the hydroxide anion. To extrapolate the energy barrier between intermediate 1 and intermediate 2 (Supplementary Table S6; coordinates in Supplementary Table S7) at the edges of the two disjunct PESs, a single-point electronic energy (EE^SP^) calculation was conducted based on intermediate 1, appended with a neutral hydrogen peroxide molecule positioned 10 Å away from the selenium center. Subsequently, another EE^SP^ calculation was conducted on intermediate 2 appended with two hydroxide anions placed 10 Å apart from each other and 10 Å away from the selenium center to minimize the interaction among the charged molecular structures. In this case, both structures have a consistent number of total electrons. Our QM computations indicate that the endothermic step 2 includes an EE_gap_ of *ca.* 300 kJ/mol when the 6-31G(d,p) basis set is used. Further HF calculations with diffuse functions suggest a larger gap (*ca.* 400 kJ/mol). As the DFT energy extrapolation at the B3LYP/6-311+G(d,p) level of theory agrees with all three HF computations and shows an EE_gap_ of 371.93 kJ/mol (Supplementary Table S6), we believe that step 2 is the most endothermic process among the four steps.

**SCHEME 1:**
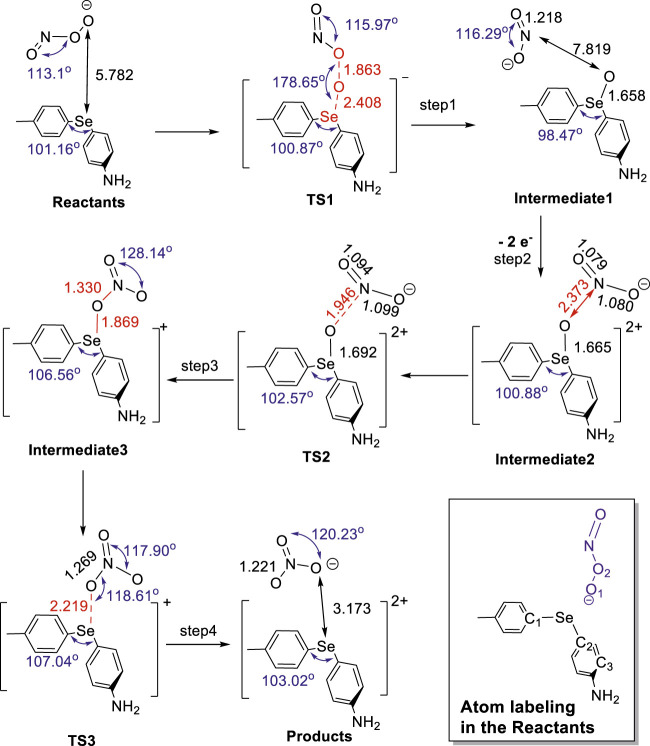
Geometry of stationary points anchored on the PES (calculated at the HF/6-311+G(d,p) level of *ab initio* theory) for the predicted four-step mechanism of the reaction converting peroxynitrite to nitrate through the catalysis of diaryl selenide in solution.

In terms of reaction thermochemistry (Figure 1), we note that at the HF/6-311+G(d,p) level of theory, the peroxy bond is broken, and the bridging oxygen in transition state 1 (TS1) is transferred to Se, releasing 157.03 kJ/mol of heat (Table 1) after overcoming an energy barrier of 99.82 kJ/mol. When comparing the overall activation energies, TS2 (step 3) illustrates the restoration of the interaction between the now selenium-bound oxygen and the nitrogen moiety, exhibiting a relatively low activation energy (14.04 kJ/mol) but being highly exothermic (−109.54 kJ/mol). In contrast, in step 4, the selenium-bound oxygen is transferred back to NO_2_ to form nitrate, which requires an activation energy of 16.04 kJ/mol. Subsequently, after the two-electron transfer step, the reaction from intermediate 2 to products is exothermic (−117.49 kJ/mol). By comparison, the activation energy for TS1 calculated at the DFT level is about half of that calculated at the HF level: 50.42/66.49 kJ/mol and −15.72/42.04 kJ/mol for TS2 (B3LYP/M06-2X; Table 1; Supplementary Table S5). These two steps are also highly exothermic at the DFT level: 181.37/177.68 kJ/mol for step 1 and 146.78/176.66 kJ/mol for step 3 (B3LYP/M06-2X; Table 1; Supplementary Table S5). From a kinetic standpoint, the transfer of an O atom from peroxynitrite to Se (step 1) appears to be the rate-limiting step. However, as the activation energy for the electron transfer step (step 2) has not been estimated, we cannot conclusively state that step 1 is overall the slowest step in the reaction. Table 1; Supplementary Table S5 list the total electronic and relative energies and the activation energy for the molecular species in Scheme 1, calculated with the B3LYP and M06-2X functionals, respectively. For reactants, TS1, and intermediate 1, the charge on the system is −1 atomic units (au) and the multiplicity is 1, while for intermediate 2 through products, the charge is +1 au and the multiplicity is 1.

Summarizing the computation results obtained with the *ab initio* HF and the DFT methods, the full reaction mechanism that requires four elementary step reactions along with values for relevant bond lengths and angles is shown in Scheme 1. In step 1, peroxynitrite reacts end-on with Se through the terminal oxygen of its peroxy bond when the terminal oxygen is transferred to the neutral diaryl selenide via the formation of a relatively high-energy activated complex (TS1). The normal mode analysis confirms the existence of only one imaginary frequency for the TS1 structure, indicating that the breaking of the peroxy bond and the formation of the Se-O bond occur simultaneously, leading to intermediate 1 [(MePh)SeO(PhNH_2_) + nitrite anion (NO_2_^−^)]. It is important to highlight that in this pseudo-S_N_2 mechanism, the active Se(II) core undergoes formal oxidation to Se(IV), simultaneously causing a reduction in the formal oxidation state of each of the two oxygen atoms in the peroxy bond from −1 to −2. After the peroxy bond is broken, the newly formed nitrite anion, although still contained “within the cage,” lacks the ability to reconnect side-on through its nitrogen atom to recover the oxygen atom initially transferred to selenium (Se) before another alteration in the oxidation state of Se takes place. Overall, we concluded from these calculations that the B3LYP and M06-2X functionals yield similar results and together validate the reaction mechanism revealed by the HF calculations, i.e., all three computational methods produce the same set of stationary points on the PES.

In step 2, the diaryl selenide oxide (MePh)SeO(PhNH_2_) is converted to a dication, [(MePh)SeO(PhNH_2_)]^2+^, after two electrons are removed. This transfer could be facilitated to a suitable oxidant (e.g., hydrogen peroxide), thereby increasing the oxidation state of Se to +6 and leading to intermediate 2 (Figure 1; Scheme 1). During step 3, Se(VI) activates its bound oxygen, inducing a transition where the oxygen relocates to form a bridge between selenium (Se) and the nitrogen atom of the nitrite anion. This transformation leads to the formation of intermediate 3 via transition state 2 (TS2; Figure 1; Scheme 1). The cleavage of the Se-O bond in step 4 results in the formation of the nitrate anion and diaryl selenide dication (products) via transition state 3 (TS3; Figure 1; Scheme 1). In the final products, the oxidation state of Se is reduced from +6 to +4, while the oxidation state of nitrogen increases from +3 (in nitrite) to +5 (in nitrate). The molecular geometry analysis of the compounds involved in the reaction shows that as the reaction proceeds from reactants to products, the Se-C2 (*p*-aniline) bond length shortens significantly from 1.92 to 1.78 Å, while the Se-C1 (*p*-tolyl) bond length only decreases slightly from 1.93 to 1.92 Å (Table 2). The C1-Se-C2 angle decreases slightly through step 1 (101.16°–100.04°; Table 2) but then increases to 106.56° in step 3 and slightly decreases to 103.02° in step 4 of the reaction. Changes in the dihedral angle C1-Se-C2-O1 point to a large rotation of the phenyl ring with respect to Se throughout the reaction and may indicate a significant substituent effect.

**TABLE 2:** Geometrical features (bond length, bond angle, and dihedral) for the chemical species appearing in the reaction mechanism of peroxynitrite isomerization optimized at the HF/6-311+G(d,p) level of theory.

Compound^[a]^	C_1_-Se^[b]^	Se-C_2_^[b]^	Se-O_1_^[b]^	O_1_-O_2_^[b]^	C_1_-Se-C_2_^[c]^	C_1_-Se-C_2_-C_3_^[c]^
Reactants	1.93	1.92	5.78	1.42	101.16	85.88
TS1	1.93	1.92	2.41	1.86	100.87	100.37
Intermediate 1	1.93	1.91	1.66	7.48	100.04	115.86
Intermediate 2	1.92	1.91	1.66	2.71	100.88	64.32
TS2	1.92	1.90	1.69	2.48	102.57	50.23
Intermediate 3	1.91	1.84	1.87	2.15	106.56	38.46
TS3	1.90	1.76	2.21	2.12	107.04	174.18
Products	1.92	1.78	3.17	2.11	103.02	177.51

[a] Atom labeling is given in Figure 1. [b] Bond lengths are in Å. [c] Bond angles and dihedrals are in degrees.

### Restoring the initial oxidation state of Se

It should be noted that at the end of peroxynitrite isomerization (Figure 1, step 4), the Se core does not end up in its initial oxidation state (Se(II)). For the catalyst to close the cycle and transform additional equivalents of peroxynitrite, selenium must be restored to its initial oxidation state by reacting with reductants in the environment. A valid visualization involves the reaction of a diaryl selenide dication (with Se(IV)) with two equivalents of hydroxide anion (e.g., produced in step 2), resulting in the production of a hydrogen peroxide/superoxide ion. In this way, the initial hydrogen peroxide, which acts as an electron acceptor, is regenerated for another reaction cycle. This shows that the reaction is not dependent on pH as the initially produced hydroxide anions are later consumed in the same reaction.

It is worth noting that certain steps in this mechanism resemble those in the peroxynitrite isomerization process catalyzed by water-soluble iron porphyrins. Although fundamentally different, the situation can certainly be compared to the well-known decomposition of peroxynitrite catalyzed by water-soluble Fe(III) porphyrins (Jensen and Riley, 2002). In that case, the net reaction is the isomerization of peroxynitrite to nitrate, but the reaction is efficiently catalyzed through an oxo-ferryl intermediate, (O = Fe(IV)-Porph). Similarly, peroxynitrite has been observed to interact with various hemoproteins, including metmyoglobin and hemoglobin (Herold et al., 2002), peroxidases (But et al., 2002), cytochrome P450s (Lin et al., 2007), and inducible NOS oxygenase (Maréchal et al., 2007); however, the specific mechanism of interaction and the resulting net reaction may vary depending on the particular hemoprotein involved. In these cases, the isomerization to nitrate, when it occurs, is initiated through the oxidation of the heme center by peroxynitrite, resulting in an oxoiron species (O = Fe (IV)) in a cage with the nitrogen dioxide radical (NO_2_^⋅^); this species rearranges to recapture the oxygen atom, ultimately returning the catalyst to its resting state and releasing nitrate. Based on known thermodynamic quantities, while strong driving forces are expected for the one-electron oxidation of Fe(III)-Porph to the oxo-ferryl species by peroxynitrite (But et al., 2002), this is not the case for diaryl selenide with higher oxidation peak potentials (an expected formal potential >1.0 V vs Ag/AgCl) (Engman et al., 1992).

**SCHEME 2:**
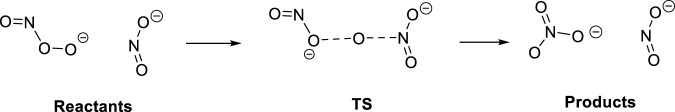
Mechanism for disproportionation reaction, in which peroxynitrite can be converted into nitrate in a concerted step in the presence of the nitrite anion.

Interestingly, while investigating the predicted mechanism shown in Figure 1, we also noted that the nitrite anion (NO_2_^−^) could catalytically interact with the surrounding peroxynitrite (OONO^−^) once it is generated from step 1. This special interaction can lead to an oxygen transfer (TS in Supplementary Figure S2; coordinates in Supplementary Tables S8–S11), convert OONO^−^ into NO_3_^−^ in one step, and regenerate NO_2_^−^ for the next reaction cycle (Scheme 2). However, despite the successful characterization of the TS, the repulsion force between two anions in both reactants and products impedes full convergence to stationary points (Supplementary Figure S2). Hence, to explore the PES and extrapolate the thermodynamic and kinetic properties of this concerted-step reaction, the surface near the anchored saddle point was studied utilizing two different methods, namely, single-point energy calculations on the proposed geometries with increasing distance between the nitrogen atoms (Supplementary Tables S12–S13; Supplementary Figure S3) and constrained geometry optimizations with a constant distance between the nitrogen atoms (Supplementary Tables S14–S15; Supplementary Figure S4), with both methods having an energy convergence to 1.0 × 10^−5^ Hartree for both reactants and products (Supplementary Tables S12–S15). When the *ab initio* HF method was applied, our computations suggested that OONO^−^ can directly deliver one O atom toward the N center in NO_2_^−^ after overcoming *ca.* 99 kJ/mol of activation energy and release 250 kJ/mol of heat (Supplementary Table S16). On the other hand, the DFT method produces a lower energy barrier (*ca.* 70 kJ/mol) and smaller ∆H (*ca.* 240 kJ/mol) at the B3LYP/6-311+G(d,p) level of theory (Supplementary Table S16). Thus, our extrapolated energetics still suggest that this disproportional reaction demands higher energy input than step 3 in the Se oxidation reaction shown in Figure 1. Considering the exothermic nature of this reaction and the regeneration of NO_2_^−^, this process sheds light on another reasonable and competitive path for isomerization. This finding indicates that the existence of NO_2_^−^ can cooperatively catalyze the conversion of OONO^−^ into NO_3_^−^, which may indirectly enhance the catalytic activity near the Se(IV) core.

**SCHEME 3:**
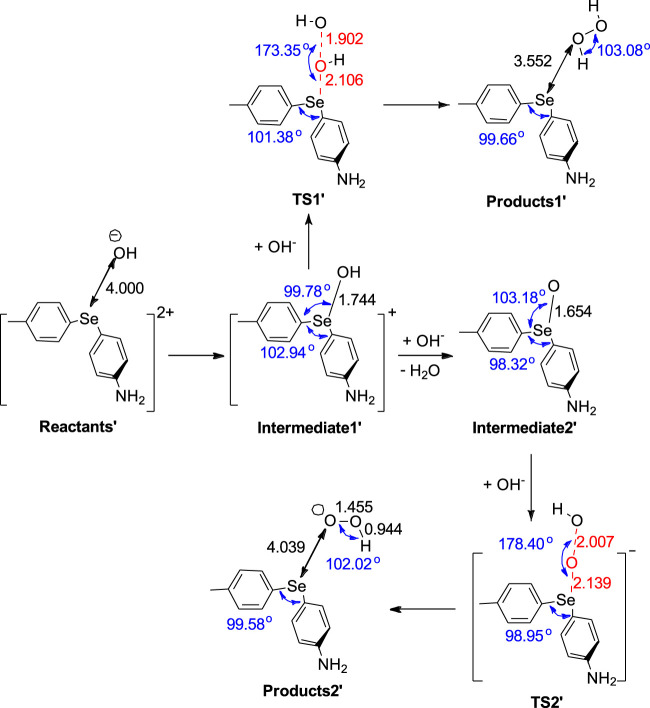
Mechanism for the reaction that restores the initial oxidation state of Se and converts [(MePh)Se(PhNH_2_)]^2+^ into (MePh)Se(PhNH_2_), generating hydrogen peroxide (H_2_O_2_)/superoxide ion (HOO^−^) as byproducts.

Although Scheme 1 and Scheme 2 show mechanistic pathways that could potentially explain the conversion of peroxynitrite to nitrate, neither pathway points to how Se reverts to its original oxidation state (+2) after peroxynitrite isomerization. Hence, the question arises as to how Se can undergo reduction from +4 to +2 to complete the catalytic cycle. Therefore, we hypothesized that this can be achieved by a reaction with reducing species (present in the environment) that can donate electrons to Se. For example, one candidate can simply be the background nitrite anion, which can escape and ultimately be subject to a hydrolytic disproportionation (But et al., 2002). In this study, we show through QM computations that another possible endogenous reducing agent could be the hydroxide anion (initially produced via hydrogen peroxide or already present at a basic pH), which, upon oxidation, would yield (regenerate) hydrogen peroxide (involved in the electron transfer from diaryl selenide). In brief, the diaryl selenide dication, [(MePh)Se(PhNH_2_)]^2+^ (Scheme 3; Supplementary Tables S17-S18), could be reduced to diaryl selenide, (MePh)Se(PhNH_2_), by reacting with one equivalent of hydroxide ion (reactants’).To start this process, one hydroxide anion will bond to Se(IV) of [(MePh)Se(PhNH_2_)]^2+^ after nitrate is released, generating [(MePh)SeOH(PhNH_2_)]^+^ (intermediate 1′). In one reaction pathway, the approach of a second equivalent of hydroxide anion can react with the already attached hydroxyl group by elongating the Se-O bond (TS1′), leading to neutral (MePh)Se(PhNH_2_) and hydrogen peroxide (products 1′), a step that restores the initial Se(II) through reduction. It should be noted that intermediate 1′ can also follow a different reaction pathway by losing a water molecule to form the neutral compound, (MePh)SeO(PhNH_2_) (intermediate 2′). Then, a second hydroxide anion can extract the oxygen atom from intermediate 2′ in a concerted step via TS2′, leading to neutral (MePh)Se(PhNH_2_) and superoxide ion (HOO^−^) (products 2′); this completes the reduction of Se to oxidation state +2 and restores the initial state of selenium (Se(II)).

### Molecular orbital analysis and electrostatic potential maps

**FIGURE 2:**
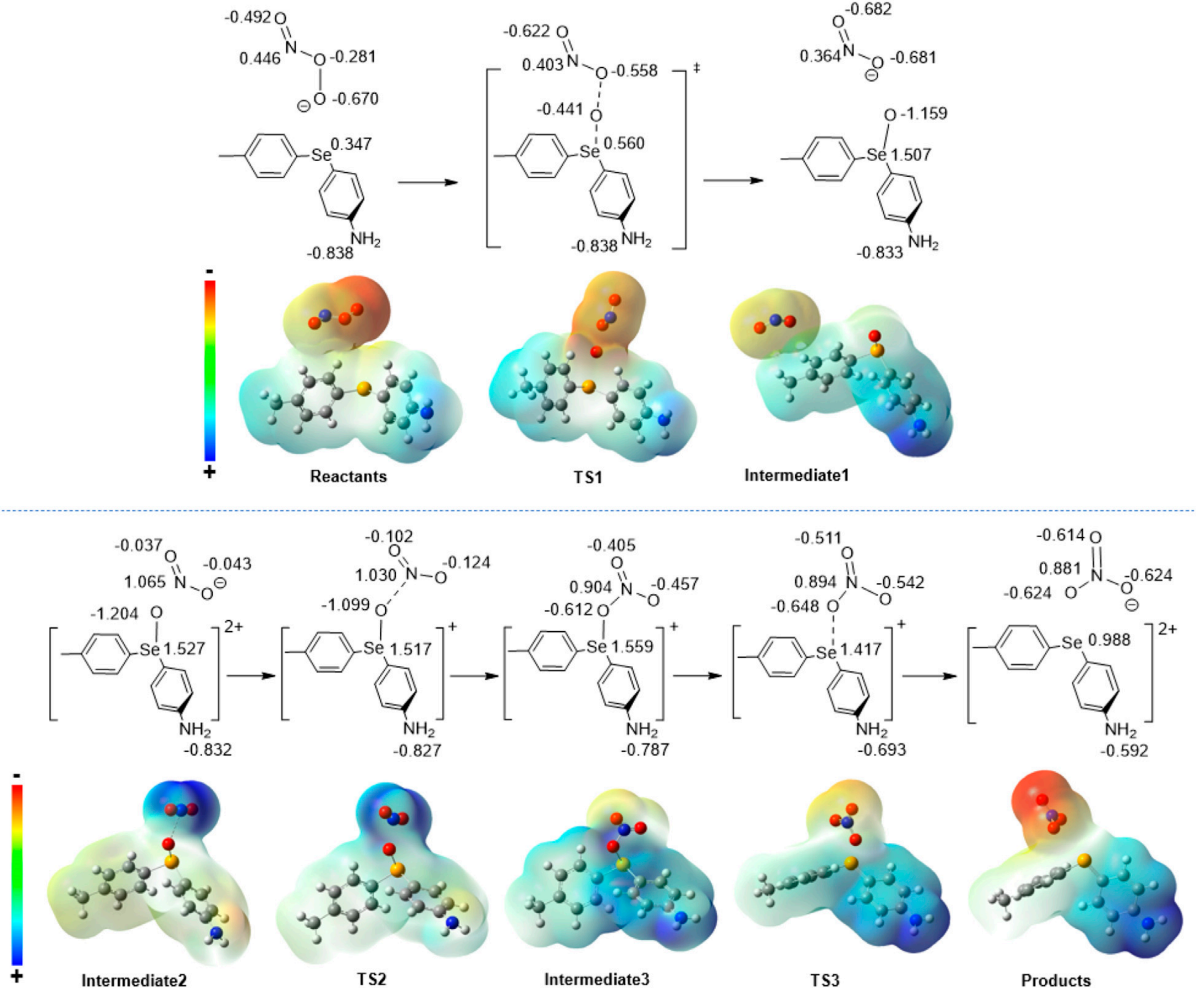
NBO atomic charges (in atomic units, abbreviated as a.u.) calculated at the HF/6-311+G(d,p) level of *ab initio* theory. ESP for the molecular species involved in the reaction of peroxynitrite with diaryl selenide. The ESP maps show how the shifts in electronic density at various steps in the reaction can be inferred from the changes in the ESP. The electronic density shifts are illustrated for the reaction step.

To gain a more comprehensive understanding of the atomic rearrangements within the transition states and intermediates along the proposed reaction path, NBO analyses were performed to explore the progression of the chemical reaction. The elucidation of these changes in partial atomic charges not only enhanced our understanding of the molecular rearrangements but also provided valuable information for unraveling the underlying mechanisms governing the behavior of the system along the entire trajectory on the PES. Figure 2 shows a visual representation of the partial atomic charges along with the associated ESP maps. Upon scrutinizing the alterations in the electronic structure of the peroxynitrite/diaryl selenide adduct throughout the course of the reaction, a notable observation arises from the NBO analysis. The charge on Se increases from 0.347 to 0.560 (a. u.) as the reaction progresses from reactants to TS1 and reaches 1.507 in intermediate 1. Correspondingly, the negative charge on the terminus O of the peroxy bond slightly decreases from −0.670 to −0.441 and then increases to −1.159 in intermediate 1, indicating that the peroxy bond in peroxynitrite within the adduct is cleaved in step 1 concomitant with the change in the oxidation states of both Se and O atoms (Se(II)→Se(IV) and O(-1)→(O(-2)). Additionally, there are significant changes in the partial atomic charges of N and the other O of the peroxy bond of peroxynitrite as the reaction progresses from reactants to intermediate 1.

**FIGURE 3:**
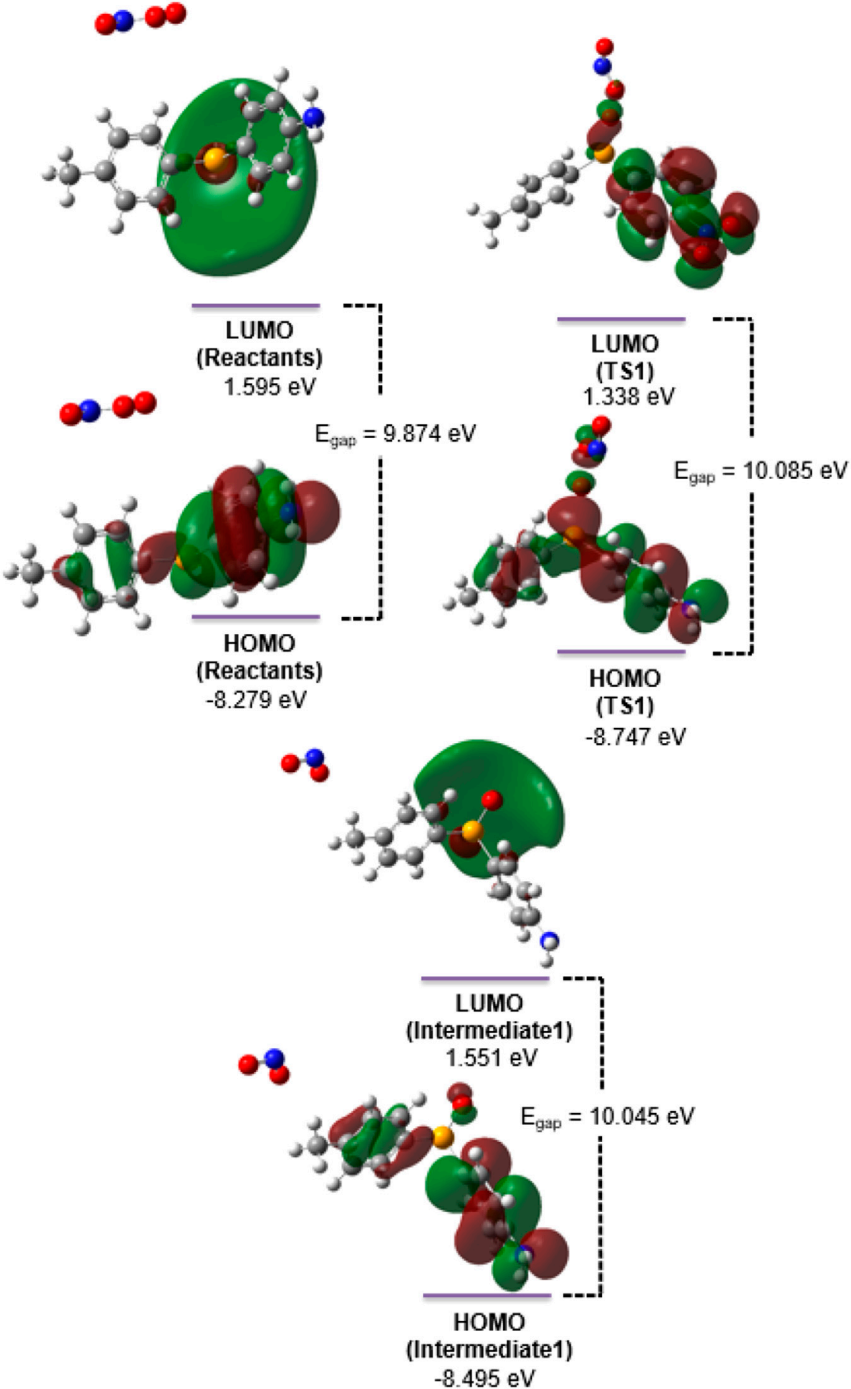
HOMO and LUMO for the molecular species (reactants, TS1, and intermediate 1) involved in step 1 of the isomerization reaction. The calculations were performed at the HF/6-311+G(d,p) level of *ab initio* theory using the PCM for solvation and water as the solvent. The molecular orbitals were plotted using the transparent isosurface with isovalue 0.02.

After losing two electrons (step 2), the nitrite moiety holds a more positive ESP in intermediate 2 but reverses to a negative ESP while the reaction progresses through intermediate 3 to the final products when the oxygen atom is delivered back to the NO_2_ moiety. The changes in ESP reveal the importance of the two-electron transfer in step 2 of the reaction moving forward, which shifts the electron density away from the nitrite anion, facilitating the cleavage of the Se-O bond and the transfer of oxygen back to the nitrite anion moiety to form a nitrate anion through TS3. To further explore the energetics of the frontier molecular orbitals (FMOs), the highest occupied molecular orbital (HOMO) and the lowest unoccupied molecular orbital (LUMO) of all stationary points (steps 1–4, Figure 1; Scheme 1) were visualized and investigated. In step 1 of the reaction, the HOMO-LUMO gap (Figure 3) increases from 9.874 eV (reactants) to 10.085 (TS1) and then decreases slightly to 10.045 eV in intermediate 1. The LUMO distribution generally encompasses a diffused orbital centered on the selenium–oxygen transfer region, while the HOMO is spread throughout the ring system, pointing to potential electronic effects with the incorporation of a variety of electron-withdrawing or -donating substituents.

**FIGURE 4:**
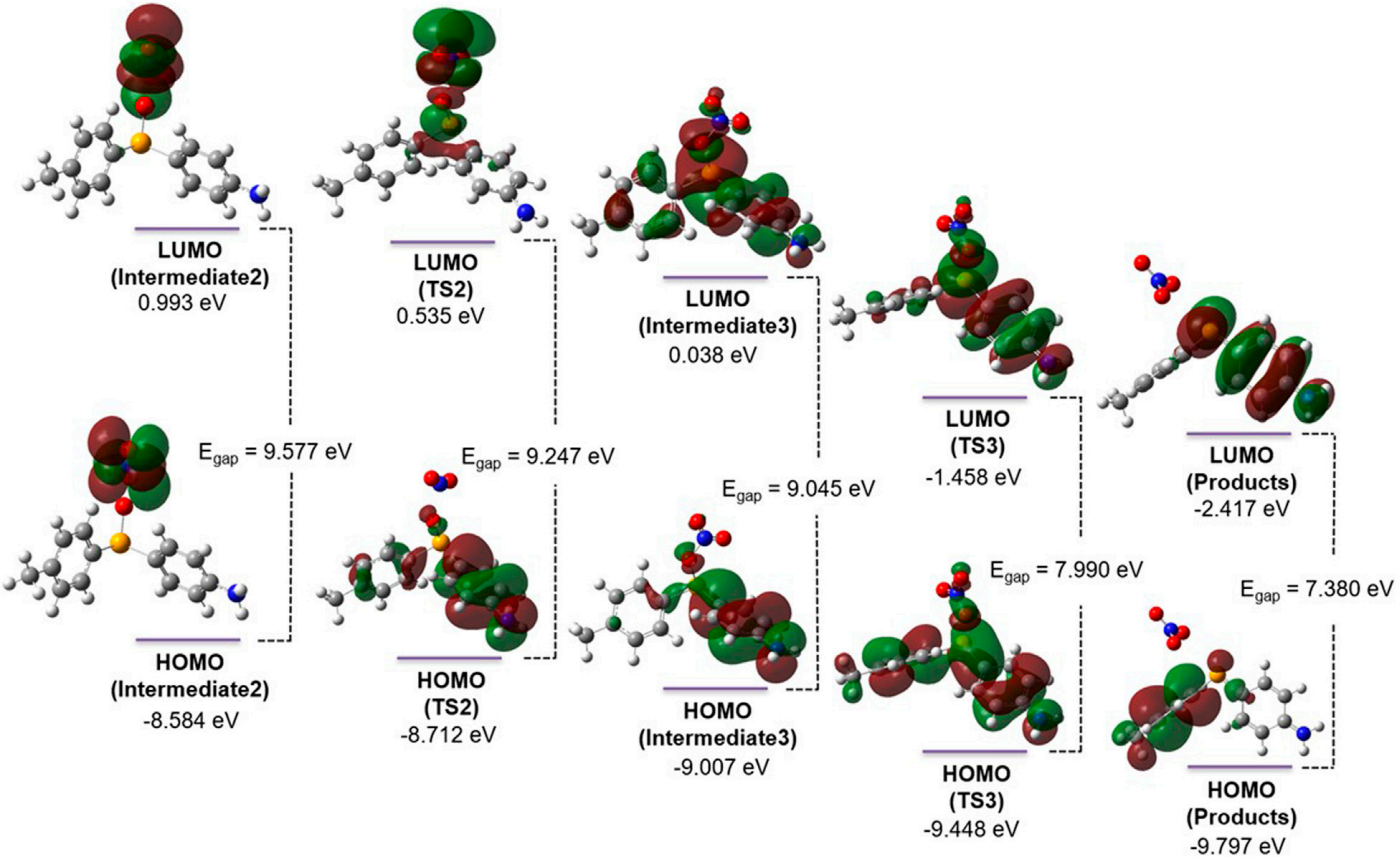
HOMOs, LUMOs, and the associated energy gaps of the molecular species involved in steps 2–4 of the reaction between peroxynitrite and diaryl selenide. The intermediates, transition states, and products were calculated at the HF/6-311+G(d,p) level of *ab initio* theory in water using the PCM for solvation. These orbitals are plotted using a transparent isosurface (isovalue of 0.02).

Additionally, the role of the two-electron transfer step in the reaction mechanism is revealed by the change in the HOMO-LUMO gap of the system. As shown in Figure 4, removing two electrons in step 2 oxidizes Se (Se(IV)→Se(VI)) and reduces the HOMO-LUMO gap from 10.045 (intermediate 1) to 9.577 eV (intermediate 2). Additionally, the HOMO and LUMO localize on the nitrite anion (intermediate 2) after electron transfer, while the LUMO extends to the whole system after the O atom moves to bridge the Se and N atoms (intermediate 3). In step 3, the LUMO begins delocalizing and extends to the π conjugation in intermediate 3 once the Se-O-N bridge is formed. The gap between the frontier orbitals shrinks further in TS3 as the electron density begins to shift to the emerging nitrate anion. Starting from this point, the HOMO-LUMO gap further decreases from 9.577 (intermediate 2) to 9.045 eV (intermediate 3) and eventually decreases to 7.380 eV (in products) in the last step of the reaction (step 4). In intermediate 3, the HOMO is localized on *p*-aniline, while in products, it is localized on *p*-tolyl. Conversely, the LUMO is extended mostly over the entire structure in intermediate 3 but is localized on *p*-aniline in products and has further implications for substituent effects in this system.

**FIGURE 5:**
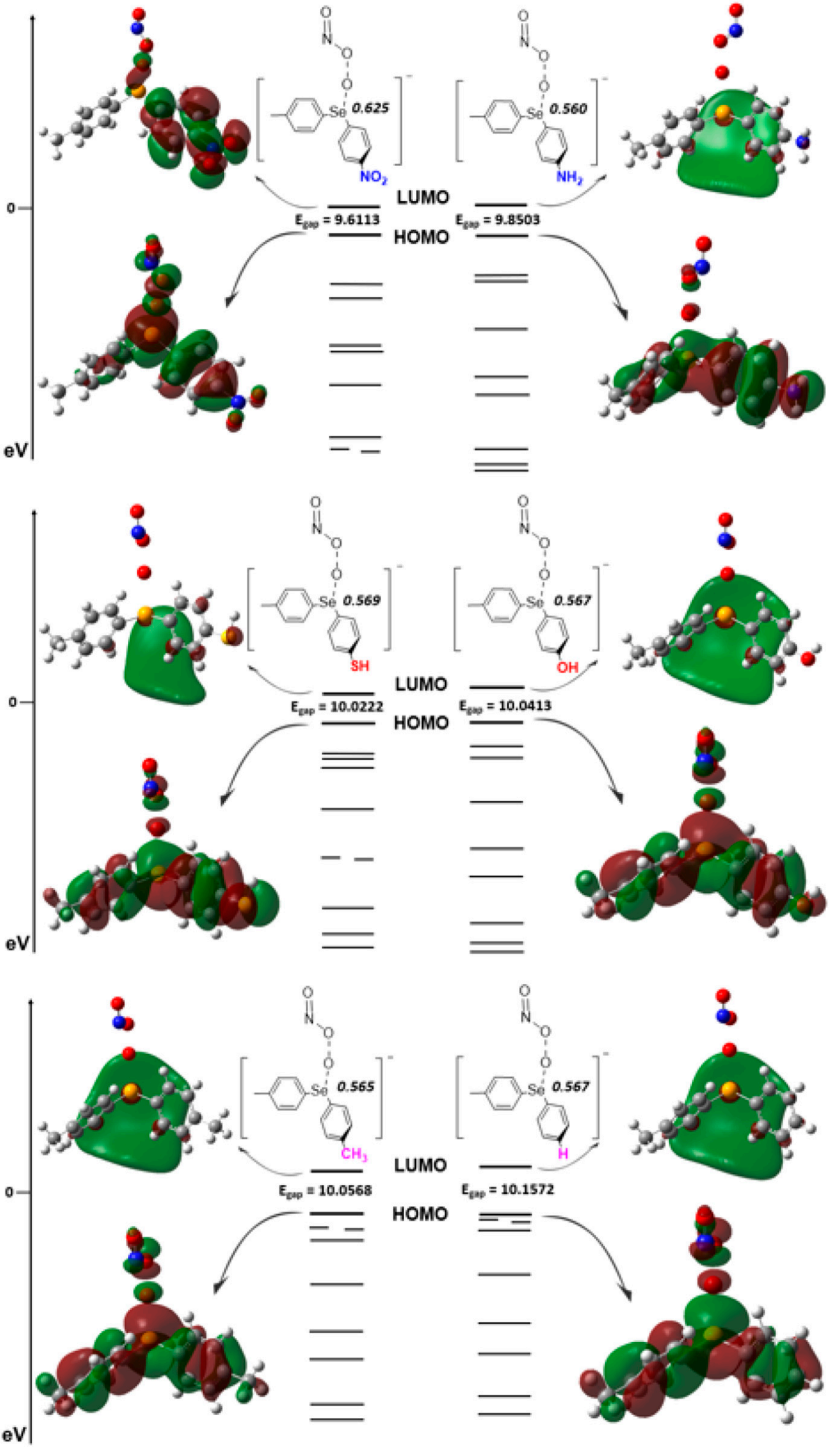
Frontier molecular orbitals along with the HOMO-LUMO gap (E_gap_) for TS1 with various substituent groups according to their electronic effects (-NO_2_, -NH/-SH, -OH/-CH_3_, and -H) in the rate-determining step of the peroxynitrite isomerization reaction showing the NBO charge at each selenium core. Computations are performed at the HF/6-311+G(d,p) level of *ab initio* theory using the PCM with water as the solvent.

Our computational results on the reaction mechanism for peroxynitrite conversion in step 1 (*vide supra*) suggest that the localization of the FMOs on the phenyl rings adjacent to Se points to a significant substituent effect for this reaction. For example, in reactants/intermediate 1, while the LUMO shows a diffused orbital encompassing the Se/ring center, the HOMO extends toward the *p*-aniline moiety. It is interesting to note that in step 1 of the reaction, the HOMO and LUMO extend over Se and the adjacent phenyl rings (Figure 4) and do not coalesce over peroxynitrite until the two-electron transfer takes place (steps 3 and 4 in Scheme 1; Figure 1). This distribution of FMOs prompted us to further explore possible electron-withdrawing and -donating substituent effects on TS1 of step 1 (Figure 5). Therefore, to further understand the role of the amino group in the initiation step (step 1), a total of 12 substituents at the *-para* position were selected to examine their impact on step 1 (Supplementary Tables S19–S22; coordinates in Supplementary Tables S23–S28). Interestingly, at the HF/6-31G(d,p) level of theory, all tested structures showed an E_a_ value of *ca.* 88 kJ/mol. This was within ±2 kJ/mol (2.3% deviation) when compared with E_a_ of step 1 with -NH_2_ as the substituent. Additionally, our computations indicated that the reaction enthalpy of step 1 with 12 different -R groups was consistently approximately −160 kJ/mol, with ±13.43 kJ/mol (8.4% deviation) compared with -NH_2_ as the substituent. Therefore, with little variation in E_a_ and reaction enthalpy, the significance of molecular orbital interactions becomes evident as they play a pivotal role in elucidating the underlying mechanism and potential impact that ring substituents may have on orbital mixing. The recognition of substantial changes in the positions and angles (Table 2) of the phenyl rings relative to selenium (Se) and each other, along with evidence from the MO analysis, prompts a vital exploration of molecular orbital overlaps, a fundamental aspect of this study. Based on the initial test of 12 different -R group substituents, a total of 6 different substituents were selected (R = -NO_2_, -NH_2_, -SH, -OH, -CH_3_, and -H) that could provide a wide scope of electron-donating and -withdrawing capabilities and placed in the *para-*position to further probe how the FMOs and the HOMO-LUMO E_gap_ could impact the oxygen transfer in step 1. The HOMO-LUMO E_gap_ was probed with an increasing trend of -NO_2_ < -NH_2_ < -SH < -OH < -CH_3_ < -H. Among these, -NO_2_ and -NH_2_ serve as both σ and π electron-withdrawing groups, while -SH and -OH act as σ withdrawing but π electron-donating groups, and -CH_3_ and -H function as σ and inductive electron-donating groups. As anticipated, the trend aligns with -CH_3_ and -H being the most significant σ and inductive electron-donating groups, resulting in the largest energy gap for electron excitation. Typically, a pronounced diffuse LUMO is evident in the LUMOs of all substituents, except for the -NO_2_ group. This distinction becomes particularly notable with the -NO_2_ substituent as the absence of this sizable orbital suggests that the potential for populating the electron density is localized away from the selenium center, pulling electron density into the orbitals on the *p-*aniline moiety’s -NO_2_-substituted ring system. Moreover, in the molecular orbitals of the LUMO for the -NO_2_-substituted ring system, there is an observable orbital mixing between selenium and oxygen atoms, indicating an eventual transfer. The lowering of the HOMO-LUMO E_gap_, along with the largest NBO charge (Figure 5) on the selenium, provides evidence that for TS1, the -NO_2_ substituent is the most potent in affecting the catalytic system. This underscores that applying more electron-withdrawing substituents at the *-para* position can better facilitate the oxygen transfer (step 1) to initiate the OONO^−^ to NO_3_^−^ conversion.

## Conclusion

In conclusion, our study highlights the intricate mechanisms underlying the conversion of peroxynitrite to nitrate facilitated by selenium-based catalysts, shedding light on the potential for effective applications in health and environmental sciences. Using a comprehensive quantum mechanical approach, we meticulously characterized the catalytic behavior of diaryl selenide and uncovered a complex redox process that unfolds in four essential steps. The critical role of electron transfer—particularly through the interaction with hydrogen peroxide—was emphasized, showcasing the necessity of a suitable electron acceptor in driving the reaction forward in solution.

Our findings also revealed a significant disproportionation pathway facilitating the conversion of peroxynitrite into nitrate. This process not only underscores the cooperative catalytic effect present in this system but also establishes a thermodynamic baseline for future investigations. Through detailed analysis of frontier molecular orbitals and electronic structures, we illustrated how selenium dynamically navigates through various oxidation states, further emphasizing the profound influence of substituent modifications on catalytic activity.

Crucially, this mechanism diverges from traditional heme- or porphyrin-catalyzed pathways, demonstrating a distinct approach that operates without radical intermediates. The insights gained from our molecular orbital analysis provide a comprehensive understanding of the electronic rearrangements throughout the reaction, revealing the importance of substituent effects in enhancing catalytic efficiency.

Ultimately, our elucidation of this catalytic mechanism not only paves the way for experimental validation but also opens avenues for harnessing this chemical transformation in therapeutic and environmental contexts, underscoring the relevance of selenium-based catalysts in future scientific explorations.

## Data Availability

The original contributions presented in the study are included in the article/Supplementary Material; further inquiries can be directed to the corresponding author.
