# Supplementary material for: Homogenous catalysis of peroxynitrite conversion to nitrate by diaryl selenide: a theoretical investigation of the reaction mechanism
Source: Front Chem. 2024 Dec 16;12:1486175. doi: 10.3389/fchem.2024.1486175 (PMC11682900; doi:10.3389/fchem.2024.1486175)
Supplement: Supplementary file 1 [file DataSheet1.PDF]

Homogenous catalysis of peroxynitrite conversion to nitrate by diarylselenide: A theoretical investigation of the reaction mechanism

Supplemental Information

Yuan Xue [1,2$], Carrie Salmon [3$], Valentin Gogonea [4,5,6*]

[1] Department of Chemistry and Biochemistry, The University of Mississippi, University, MS 38677

[2] Department of Chemistry and Biochemistry, Oberlin College and Conservatory, Oberlin, OH 44074

[3] Department of Physics, Kent State University, Kent, OH 44242

[4] Department of Chemistry, Cleveland State University, 2399 Euclid Ave, Cleveland, OH 44115

[5] Department of Cardiovascular & Metabolic Sciences, Lerner Research Institute, Cleveland Clinic, Cleveland, OH 44195

[6] Center for Microbiome & Human Health, Cleveland Clinic, Cleveland OH 44195

[$] Contributed equally.

*** Correspondence:**
Valentin Gogonea: v.gogonea@csuohio.edu

Keywords: diarylselenide, peroxynitrite, nitrate, nitrite, reaction mechanism, substituent effect, potential energy surface, frontier molecular orbitals, and electrostatic potential map, NBO analysis.

Table of Contents

	Section	Page
Oxidation	**Table S1.** The electronic energy (in Hartree) determined at B3LYP/6-311+G(d,p) level of theory for the optimized geometries with constrained distance between the active oxygen connected to Se and N in NO_2^- ($R(O^{Se}$-N, in Å). The fully characterized TS2 is also illustrated along with the constrained geometries.	**S4**
	**Figure S1.** The nature of the PES probed near the fully characterized TS2 at B3LYP/6-311+G(d,p) level of theory. Detailed energetics and distances are illustrated in Table S1.	**S5**
	**Table S2.** Cartesian coordinates (in Angstrom, Å) of characterized stationary points on the Se oxidation reactions at B3LYP/6-311+G(d,p) level of theory.	**S6**
	**Table S3.** Cartesian coordinates (in Angstrom, Å) of characterized stationary points on the Se oxidation reactions at HF/6-311+G(d,p) level of theory.	**S12**
	**Table S4.** Cartesian coordinates (in Angstrom, Å) of characterized stationary points on the Se oxidation reactions at M062-2X/6-31+G(d,p) level of theory.	**S20**
	**Table S5.** Electronic energies (EE), relative energies (ΔE), and the activation energies (Ea) for stationary points anchored on the potential energy surface at M06-2X/6-31+G(d,p) level of theory.	**S26**
	**Table S6.** Single point energy extrapolated at different levels of theory to estimate the thermodynamic aspect of Step 1 and Step 3.	**S26**
	**Table S7.** Cartesian coordinates (in Angstrom, Å) of the two proposed stationary points (based on Int1 and Int2) in step 2.	**S27**
NO_2^- + $ONOO^-$ = NO_2^- + NO_3^-	**Figure S2.** A rough estimation of the potential energy surface near the oxygen-transfer transition state for the reaction converting NO_2^- and $ONOO^-$ to NO_2^- and NO_3^-	**S29**
	**Table S8.** Cartesian coordinates (in Angstrom, Å) of the oxygen transfer TS to convert NO_2^- and $ONOO^-$ to NO_2^- and NO_3^- at HF/6-31G(d,p) level of theory.	**S29**
	**Table S9.** Cartesian coordinates (in Angstrom, Å) of the oxygen transfer TS to convert NO_2^- and $ONOO^-$ to NO_2^- and NO_3^- at HF/6-31+G(d,p) level of theory.	**S29**
	**Table S10.** Cartesian coordinates (in Angstrom, Å) of the oxygen transfer TS to convert NO_2^- and $ONOO^-$ to NO_2^- and NO_3^- at HF/6-311+G(d,p) level of theory.	**S30**
	**Table S11.** Cartesian coordinates (in Angstrom, Å) of the oxygen transfer TS to convert NO_2^- and $ONOO^-$ to NO_2^- and NO_3^- at B3LYP/6-311+G(d,p) level of theory.	**S30**
	**Table S12.** The electronic energy (in Hartree) determined for single-point energy calculation determined on the proposed structures to convert NO_2^- and $ONOO^-$ to NO_2^- and NO_3^-	**S30**
	**Table S13.** The relative energy (in kJ/mol) determined for single-point energy calculation determined on the proposed structures to convert NO_2^- and $ONOO^-$ to NO_2^- and NO_3^-	**S31**
	**Figure S3.** The potential energy surface for the reaction converting NO_2^- and $ONOO^-$ to NO_2^- and NO_3^- based on single-point energy calculation determined on the proposed structures	**S31**
	**Table S14.** The electronic energy (in Hartree) determined by constrained geometry optimization with frozen N…N distances to convert NO_2^- and $ONOO^-$ to NO_2^- and NO_3^-	**S32**
	**Table S15.** The relative energy (in kJ/mol) determined by constrained geometry optimization with frozen N…N distances to convert NO_2^- and $ONOO^-$ to NO_2^- and NO_3^-	**S32**
	**Figure S4.** The potential energy surface for the reaction converting NO_2^- and $ONOO^-$ to NO_2^- and NO_3^- based constrained geometry optimization with frozen N…N distances	**S33**

Table S16. A summarized E_a and ΔH (in kJ/mol) for the reaction of converting NO_2^- and $ONOO^-$ to NO_2^- and NO_3^- **S33**

Reduction

Table S17. Cartesian coordinates (in Angstrom, Å) of characterized stationary points on PES (Depicted in Scheme 3 in manuscript) calculated at HF/6-31G(d,p) level of theory. **S34**

Table S18. Total electronic energies for the compounds involved in the restoration of the initial oxidation state of Se on diarylselenide calculated at the HF/6-31G(d,p) level of theory. **S40**

Table S19. The electronic energy (in Hartree) of fully characterized stationary points on the PES for reaction step 1 with different -R group calculated at HF/6-31G(d,p) level of theory **S41**

Table S20. A summarized E_a and ΔH (in kJ/mol) for the reaction step 1 with different -R group calculated at HF/6-31G(d,p) level of theory **S41**

Table S21. The electronic energy (in Hartree) of additional fully characterized stationary points on the PES for reaction step 1 with different -R group calculated at HF/6-31G(d,p) level of theory **S41**

Table S22. A summarized E_a and ΔH (in kJ/mol) for the reaction step 1 with additional systems with different -R group calculated at HF/6-31G(d,p) level of theory **S41**

-R Effect

Table S23. Cartesian coordinates (in Angstrom, Å) of the TS in the Se oxidation reaction step 1 with $-NO_2$ as the -R group at the *para* position calculated at HF/6-311+G(d,p) level of theory. **S42**

Table S24. Cartesian coordinates (in Angstrom, Å) of the TS in the Se oxidation reaction step 1 with $-NH_2$ as the -R group at the *para* position calculated at HF/6-311+G(d,p) level of theory. **S43**

Table S25. Cartesian coordinates (in Angstrom, Å) of the TS in the Se oxidation reaction step 1 with -SH as the -R group at the *para* position calculated at HF/6-311+G(d,p) level of theory. **S43**

Table S26. Cartesian coordinates (in Angstrom, Å) of of the TS in the Se oxidation reaction step 1 with -OH as the -R group the *para* position calculated at HF/6-311+G(d,p) level of theory. **S44**

Table S27. Cartesian coordinates (in Angstrom, Å) of the TS in the Se oxidation reaction step 1 with -H as the -R group the *para* position calculated at HF/6-311+G(d,p) level of theory. **S45**

Table S28. Cartesian coordinates (in Angstrom, Å) of the TS in the Se oxidation reaction step 1 with $-CH_3$ as the -R group the *para* position calculated at HF/6-311+G (d,p) level of theory. **S46**

Table S1. The electronic energy (in Hartree) determined at B3LYP/6-311+G(d,p) level of theory for the optimized geometries with constrained distance between the active oxygen connected to Se and N in NO_2^- (R(O^{Se}…N, in Å).

Scan Step	R(O^{Se}…N)	Scan Step Size[a]	Electronic Energy	ΔEE[b]
**1**	2.910	-	-3239.775092	-
**2**	2.810	0.100	-3239.776473	-0.00138
**3**	2.760	0.050	-3239.777235	-0.00076
**4**	2.710	0.050	-3239.778042	-0.00081
**5**	2.660	0.050	-3239.778907	-0.00086
**6**	2.655	0.005	-3239.778956	-0.00005
**7**	2.648	0.007	-3239.779086	-0.00013
**8**	2.642	0.006	-3239.779199	-0.00011
**9**	2.635	0.007	-3239.779331	-0.00013
**10**	2.628	0.007	-3239.779466	-0.00013
**11**	2.621	0.007	-3239.779601	-0.00014
**12**	2.614	0.007	-3239.776469	**0.00313**
**TS2[c]**	2.614	-	-3239.776469	-
**13**	2.607	0.007	-3239.779872	-0.00340
**14**	2.600	0.007	-3239.780015	-0.00014
**15**	2.560	0.040	-3239.780862	-0.00085
**16**	2.510	0.050	-3239.782012	-0.00115
**17**	2.460	0.050	-3239.783867	-0.00185
**18**	2.410	0.050	-3239.785694	-0.00183
**19**	2.310	0.100	-3239.789734	-0.00404
**20**	2.210	0.100	-3239.794105	-0.00437
**21**	2.110	0.100	-3239.798902	-0.00480
**22**	2.010	0.100	-3239.803791	-0.00489
**23**	1.910	0.100	-3239.808675	-0.00488
**24**	1.810	0.100	-3239.813424	-0.00475
**25**	1.710	0.100	-3239.818014	-0.00459
**Int 3***	1.353	-	-3239.833766	-

[a]Scan step size (ΔR, in Å) = R(O^{Se}…N)$_n$ - R(O^{Se}…N)$_{n-1}$ where n = 2 -25.

[b]The electronic energy change (ΔEE, in Hartree) = E_n - E_{n-1} where n = 2 -25. *Reported structures are fully optimized with no geometry constraint. The nature of the stationary point is further verified by harmonic vibrational frequency.

[c]The fully characterized TS2 is also illustrated along with the constrained geometries. This structure was optimized without geometry constraint and verified with frequency calculation.

Additional notes:

The only Δ**EE** with a positive value along the reaction coordinate (along the scan) is near TS2 ±0.007 Å (**Scan step 12 in Table S1**). This suggests that despite the fully characterized TS2, the Int 2 characterized at HF/6-311+G(d,p) level vanishes at B3LYP/6-311+G(d,p) level of theory and might not be able to be identified using the default searching algorithm.

Figure S1. The nature of the PES probed near the fully characterized TS2 at B3LYP/6-311+G(d,p) level of theory.

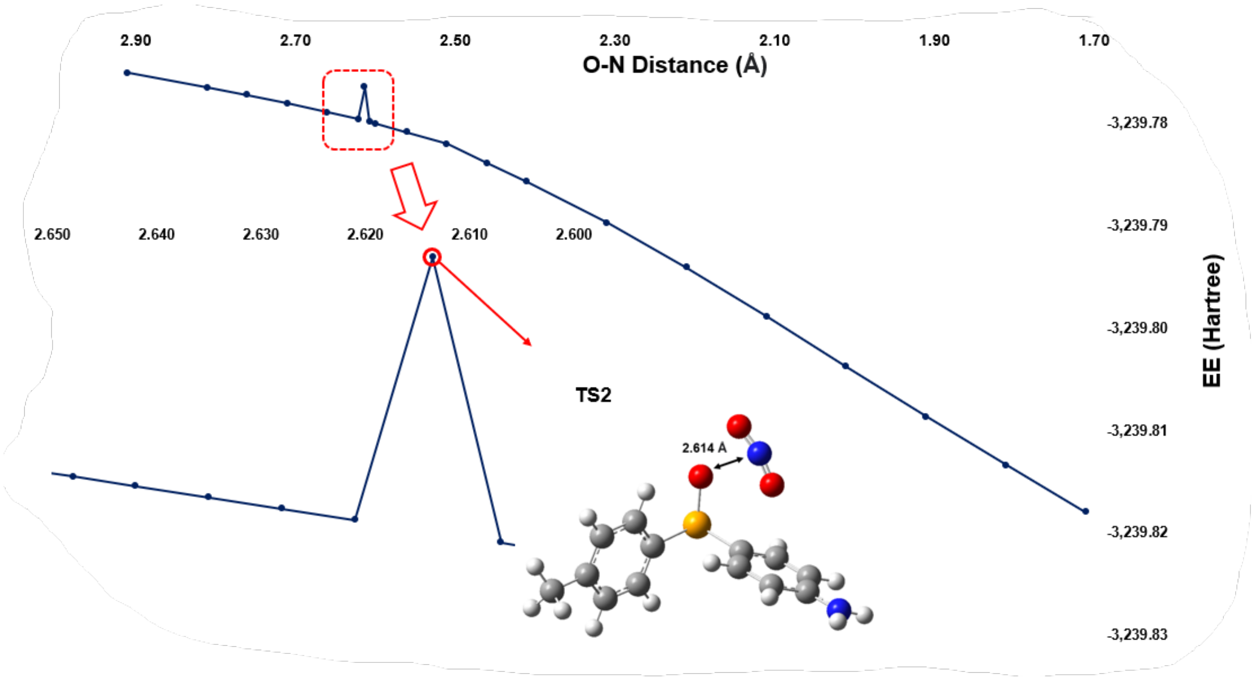

Additional notes:

This figure was made using data from Table S1. The positive ΔEE is near TS2 ±0.007 Å (**Scan step 12 in Table S1**) could be clearly probed when a constrained geometry optimization is applied. The scanning results reported herein suggest that despite the fully characterized TS2, the Int 2 characterized at HF/6-311+G(d,p) level vanishes at B3LYP/6-311+G(d,p) level of theory.

Table S2. Cartesian coordinates (in Angstrom, Å) of characterized stationary points on PES calculated at B3LYP/6-311+G(d,p) level of theory.

Compound & n_i	Structure	Atom coordinates			
Reactants<br>$n_i = 0$	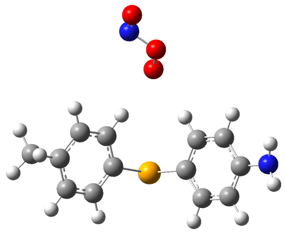	C	-3.33161200	-1.55076000	1.18755400
		C	-2.63737200	-2.72782700	0.88900300
		H	-2.99148800	-3.67470000	1.28425200
		C	-1.49073800	-2.70721400	0.09290000
		H	-0.96490600	-3.63121500	-0.11784200
		C	-1.02357400	-1.49973200	-0.42915200
		C	-1.71343800	-0.31397400	-0.15657700
		H	-1.37305200	0.63168300	-0.57198900
		C	-2.84952200	-0.34900600	0.65122700
		H	-3.37863300	0.57640500	0.85796200
		C	-4.55177900	-1.56807900	2.07600200
		H	-4.28718800	-1.30747600	3.10658400
		H	-5.01619900	-2.55609900	2.09592700
		H	-5.29788500	-0.84373600	1.74054100
		Se	0.54579700	-1.49442500	-1.59028000
		C	3.82264900	0.49695100	1.23782800
		C	2.84732600	1.28693400	0.60391000
		H	2.85135300	2.36214700	0.75323000
		C	1.88274700	0.71178400	-0.21758600
		H	1.13916600	1.34727300	-0.69090800
		C	1.86021300	-0.67364100	-0.41501000
		C	2.82534600	-1.46686800	0.21304500
		H	2.82018100	-2.54147800	0.07183000
		C	3.79745500	-0.89188200	1.02612000
		H	4.54093000	-1.52127200	1.50450100
		N	4.82610900	1.08210200	2.00536200
		H	4.61302400	1.99132300	2.39115000
		H	5.27240600	0.47791100	2.68114800
		N	-2.19555700	4.09136800	-0.44160100
		O	-2.35934100	4.94373600	0.40629400
		O	-0.90491200	3.70456600	-0.49999700
		O	-0.67635700	2.70235600	-1.47364400

**TS1**<br><br>**n_i = 1**	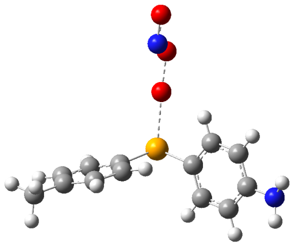	C	-3.87355200	-1.34626100	0.87855100
		C	-2.60781600	-1.41132300	1.47337600
		H	-2.51173300	-1.79550200	2.48424200
		C	-1.46310100	-0.98417900	0.80145900
		H	-0.49957400	-1.03958600	1.29368600
		C	-1.56298400	-0.48569400	-0.49897800
		C	-2.81908800	-0.41446500	-1.10969000
		H	-2.91646700	-0.03267600	-2.12059300
		C	-3.95627200	-0.83551300	-0.42257600
		H	-4.92298100	-0.76940700	-0.91190100
		C	-5.10308700	-1.83572900	1.60482200
		H	-4.99838600	-1.71960400	2.68596200
		H	-5.27949800	-2.89867700	1.40628400
		H	-5.99578500	-1.29255700	1.28660800
		Se	-0.04149400	0.21095800	-1.48537600
		C	3.60009700	-1.94773800	0.67324600
		C	3.32672800	-0.58660300	0.89197300
		H	3.96525900	-0.01328200	1.55632300
		C	2.24563400	0.02958300	0.26974100
		H	2.02783400	1.07841600	0.43600400
		C	1.41611200	-0.69553700	-0.58800000
		C	1.68738900	-2.04759000	-0.82339900
		H	1.05849700	-2.62244000	-1.49362600
		C	2.76120800	-2.66888300	-0.19683200
		H	2.96003700	-3.71903500	-0.38434500
		N	4.71017300	-2.55309700	1.25187800
		H	5.07204400	-2.11059000	2.08493600
		H	4.67353000	-3.55905400	1.33715400
		N	0.31135300	4.13861300	1.13008900
		O	0.53357100	5.26465100	1.57343900
		O	0.62803700	4.00957000	-0.11348600
		O	0.30335900	2.42608000	-0.65418800

**Int 1**<br><br>**n$_i$ = 0**	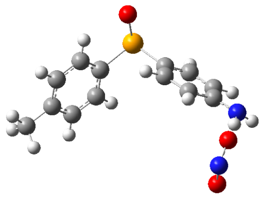	C	-4.11238700	-0.81767400	0.62692800
		C	-3.78601600	0.24320400	-0.22350700
		H	-4.37939800	1.15121700	-0.19819300
		C	-2.70960300	0.15534000	-1.10979800
		H	-2.46007400	0.97449100	-1.77459400
		C	-1.94828300	-1.00420100	-1.13561100
		C	-2.25536700	-2.08438500	-0.30858000
		H	-1.66657000	-2.99505500	-0.33706200
		C	-3.33111000	-1.98226500	0.56816000
		H	-3.56977900	-2.82042400	1.21480400
		C	-5.27554000	-0.72510000	1.58373500
		H	-5.79515400	0.22950700	1.48642000
		H	-4.93935500	-0.82371400	2.62030500
		H	-5.99852200	-1.52579300	1.40218500
		Se	-0.46719500	-1.14366500	-2.42350800
		C	3.22973900	-0.86816700	0.55265700
		C	2.20390200	0.10550600	0.60363700
		H	2.28161700	0.91508300	1.32064300
		C	1.11072200	0.03006100	-0.24307700
		H	0.33568400	0.78594900	-0.18174700
		C	1.00898700	-1.01650000	-1.16544200
		C	2.01451300	-1.98348100	-1.23601000
		H	1.94692700	-2.79659000	-1.95123800
		C	3.11003500	-1.91523300	-0.38452300
		H	3.88548300	-2.67169100	-0.44153900
		N	4.32772600	-0.76092900	1.36878200
		H	4.26580000	-0.16114100	2.19536900
		H	4.91052900	-1.57821500	1.47087800
		N	3.70978700	1.11433700	4.73367600
		O	3.49402300	-0.05118800	5.13296900
		O	4.14497800	1.22866500	3.55447300
		O	-0.53095600	0.35609800	-3.21320800

**Int 2***<br><br>**n$_i$ = 0**	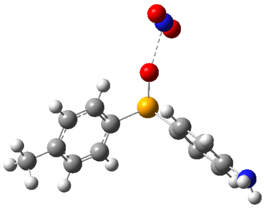	*This structure could not be optimized at B3LYP/6-311+G(d,p) level of theory. The reported structure was fully optimized at HF/6-311+G(d,p) level of theory.
**TS2**<br><br>**n$_i$ = 1**	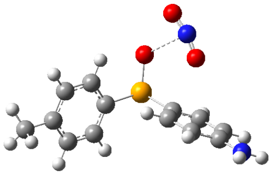	C 4.10840700 -0.90364000 -0.54797900<br>C 3.48965600 -1.51405300 0.55308200<br>H 3.94057700 -2.39259800 1.00217100<br>C 2.30816900 -1.00832000 1.08800600<br>H 1.85103400 -1.49290400 1.94397000<br>C 1.73322500 0.12053600 0.50567600<br>C 2.32979400 0.75885800 -0.57353700<br>H 1.87498100 1.64976500 -0.99076100<br>C 3.51451800 0.23897900 -1.09604900<br>H 3.98335000 0.73412100 -1.93973100<br>C 5.37550100 -1.47726100 -1.13124900<br>H 5.90327600 -0.74014500 -1.73884200<br>H 5.15067500 -2.33635900 -1.77212300<br>H 6.05071100 -1.82622400 -0.34656800<br>Se 0.11028300 0.89026300 1.28469100<br>C -3.16993200 -2.11910500 -0.38954300<br>C -3.37910400 -1.35990300 0.79028400<br>H -4.31024700 -1.46633500 1.33479300<br>C -2.40304700 -0.50000800 1.24934900<br>H -2.57735500 0.06319100 2.15951600<br>C -1.19733900 -0.34964300 0.54002200<br>C -0.96974200 -1.11319400 -0.61934800<br>H -0.03490900 -1.02499200 -1.15877500<br>C -1.94080200 -1.97505100 -1.08350300<br>H -1.76571600 -2.55367600 -1.98320300<br>N -4.12324300 -2.96488500 -0.84263100<br>H -4.98916400 -3.09136300 -0.34353200<br>H -3.97621200 -3.52697800 -1.66570100<br>N -1.99686500 2.51219600 -1.41929000<br>O -1.40871800 3.47289600 -1.53824200<br>O -2.33004600 1.47495400 -1.09963600<br>O -0.11194700 2.31655100 0.38056100

**Int 3**<br><br>**n_i = 0**	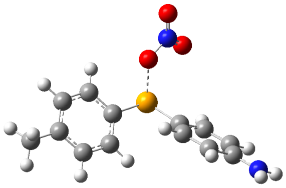	C -4.06996200 -1.31434000 0.19307100<br>C -2.95616400 -2.10671000 -0.11241300<br>H -3.05710200 -3.18519300 -0.14972500<br>C -1.71548200 -1.53971500 -0.38044100<br>H -0.88034600 -2.17542700 -0.64039200<br>C -1.58873100 -0.14729100 -0.33651400<br>C -2.69091400 0.67382800 -0.07062800<br>H -2.59511900 1.75129500 -0.06079100<br>C -3.91505400 0.08062500 0.20362400<br>H -4.76712800 0.71333200 0.42411900<br>C -5.39928800 -1.94086000 0.51509900<br>H -6.22574900 -1.31767100 0.16825500<br>H -5.50918700 -2.05594500 1.59888900<br>H -5.49250300 -2.93115300 0.06675100<br>Se 0.03807600 0.69725900 -0.90151000<br>C 3.59065700 -1.97283400 0.39742200<br>C 3.67688100 -1.08145800 -0.71335500<br>H 4.60168100 -1.01694600 -1.27281200<br>C 2.60392000 -0.31320300 -1.06397800<br>H 2.68317400 0.36420600 -1.90588300<br>C 1.37789200 -0.42595200 -0.34610600<br>C 1.29440300 -1.28584900 0.78413300<br>H 0.38769200 -1.33130400 1.37188200<br>C 2.37210500 -2.04232300 1.14319700<br>H 2.31697200 -2.69465100 2.00616300<br>N 4.63658500 -2.72268300 0.74687400<br>H 5.50728700 -2.68139700 0.23740700<br>H 4.59232200 -3.35373500 1.53351200<br>N 0.66470600 3.13885300 0.66057000<br>O 0.51376900 3.94440300 1.55712900<br>O 1.50023300 3.18460100 -0.22829600<br>O -0.19490300 2.09488400 0.68906700
**TS3***<br><br>**n_i = 1**	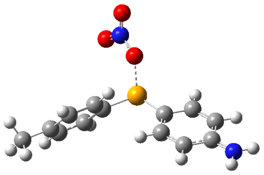	This structure could not be optimized at B3LYP/6-311+G(d,p) level of theory. The reported structure was fully optimized at HF/6-311+G(d,p) level of theory.

**Product**<br><br>**n_i = 0**	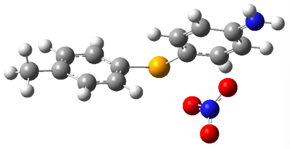	C	4.22328300	0.95865400	0.57623300
		C	4.30715800	-0.43979100	0.49481000
		H	5.23710000	-0.93687300	0.74649900
		C	3.21956200	-1.20475300	0.09384700
		H	3.30650900	-2.28271100	0.03606600
		C	2.01723700	-0.56059600	-0.21890100
		C	1.90838400	0.83522300	-0.16826900
		H	0.99066000	1.33135200	-0.45966200
		C	3.01277000	1.57733700	0.23155100
		H	2.93518200	2.65815700	0.26379500
		C	5.40282600	1.77241600	1.03435600
		H	5.36571300	2.78753300	0.63588600
		H	6.34489000	1.31001000	0.73380400
		H	5.40902000	1.84649900	2.12742200
		Se	0.58677700	-1.63919900	-0.89687000
		C	-3.25353000	-0.39566700	1.35883800
		C	-1.97517400	0.04802100	1.87518200
		H	-1.95468600	0.63749900	2.78312100
		C	-0.83262700	-0.28885900	1.23667600
		H	0.12306000	0.02788500	1.62872300
		C	-0.88538600	-1.09356100	0.04840900
		C	-2.14977100	-1.58947000	-0.43478200
		H	-2.17547600	-2.21962600	-1.31502300
		C	-3.29756000	-1.24452000	0.18717800
		H	-4.25726100	-1.59147100	-0.17394900
		N	-4.35763300	-0.09630700	1.99403800
		H	-4.35198800	0.48308000	2.82551400
		H	-5.26245900	-0.40966800	1.66172400
		N	-2.30101000	2.02476900	-1.06513400
		O	-2.39047000	3.09490600	-1.68518800
		O	-3.16869800	1.71252200	-0.20615600
		O	-1.34498300	1.23573000	-1.28283700

Table S3. Cartesian coordinates (in Angstrom, Å) of characterized stationary points on PES calculated at HF/6-311+G(d,p) level of theory.

Compound & n_i	Structure	Atom coordinates			
**Reactants** $n_i = 0$	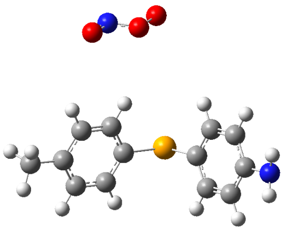	C	2.17794100	2.84684100	1.04142900
		C	1.09845700	3.56403400	0.54288600
		H	0.98293000	4.60294200	0.79849400
		C	0.15702500	2.96232000	-0.28164800
		H	-0.67018800	3.54046500	-0.65074900
		C	0.27901200	1.62423000	-0.62365200
		C	1.36021900	0.89860400	-0.13552500
		H	1.47664900	-0.13737300	-0.39608000
		C	2.29136900	1.50343700	0.69042200
		H	3.11789700	0.92332500	1.06269800
		C	3.21645900	3.50454600	1.91856800
		H	3.53552900	2.83723900	2.71172200
		H	2.83055000	4.41155400	2.36893600
		H	4.09571100	3.76872300	1.33703600
		Se	-1.01150000	0.80014700	-1.79712400
		C	-3.77588000	-1.50191800	1.27287000
		C	-2.71826900	-2.15266800	0.63921000
		H	-2.53194100	-3.19267900	0.84231800
		C	-1.90673600	-1.47193600	-0.25062100
		H	-1.09758200	-1.99682700	-0.72457200
		C	-2.12431200	-0.12902300	-0.53305300
		C	-3.18551600	0.51621300	0.09205000
		H	-3.38158300	1.55254000	-0.11495800
		C	-3.99962900	-0.15504900	0.98475700
		H	-4.81507600	0.36372900	1.45764400
		N	-4.62633000	-2.19422700	2.12660200
		H	-4.22412500	-2.99867700	2.55632600
		H	-5.10102000	-1.62709800	2.79487800
		N	4.05960300	-2.62548400	0.14028700
		O	4.40975500	-2.25907600	1.20196400
		O	2.95673500	-3.26055200	0.16944500
		O	2.57231800	-3.68834400	-1.12533600

**TS1**<br><br>**n$_i$ = 1**	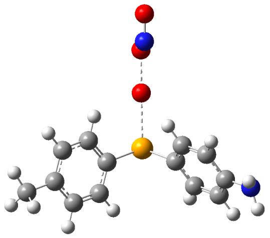	C	-3.95969400	-1.08287000	0.69557500
		C	-3.30433500	-1.96573200	-0.16369400
		H	-3.71983000	-2.94288900	-0.34100000
		C	-2.13252300	-1.60471200	-0.80032600
		H	-1.65262400	-2.30035400	-1.46469900
		C	-1.57608700	-0.34748500	-0.58132100
		C	-2.21732300	0.53943100	0.26518700
		H	-1.79788800	1.51300100	0.43488600
		C	-3.40138100	0.17059800	0.89508500
		H	-3.88614600	0.87362600	1.54969800
		C	-5.24091500	-1.49349800	1.38135900
		H	-5.64269300	-0.68329200	1.97790500
		H	-5.07225300	-2.34384300	2.03541300
		H	-5.99236600	-1.78437300	0.65374800
		Se	0.04298700	0.18265100	-1.48251400
		C	3.29604900	-2.30679100	0.87037300
		C	2.67096100	-1.21076000	1.46946100
		H	2.94106000	-0.92444500	2.47085600
		C	1.71693800	-0.48524900	0.78387900
		H	1.25701300	0.36001800	1.26162700
		C	1.34911700	-0.83680500	-0.51052700
		C	1.97139600	-1.92411200	-1.10754600
		H	1.70596900	-2.21446200	-2.10786800
		C	2.93576700	-2.65114800	-0.43032200
		H	3.40778100	-3.48943200	-0.91201200
		N	4.29819200	-3.00072400	1.53805400
		H	4.24738500	-2.95576600	2.53251500
		H	4.43804600	-3.93691000	1.22571700
		N	0.88813100	4.26986600	1.03115400
		O	1.08869200	5.37535700	1.46926300
		O	0.70330000	4.19685000	-0.18213000
		O	0.40373600	2.44074800	-0.72814600

**Int 1**<br><br>**n$_i$ = 0**	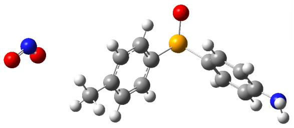	C	-2.90549700	0.35047600	0.99924200
		C	-2.60094000	-0.41170700	-0.12886200
		H	-3.36927300	-1.00167400	-0.59702700
		C	-1.32686200	-0.41497100	-0.66280800
		H	-1.11766500	-1.00484900	-1.53814000
		C	-0.33073500	0.34371300	-0.06274200
		C	-0.61175500	1.11685100	1.04588600
		H	0.15977900	1.71612900	1.49071300
		C	-1.89742200	1.11390300	1.57175700
		H	-2.11083500	1.71854400	2.43547700
		C	-4.29686300	0.32796000	1.58463200
		H	-4.45285100	1.16439400	2.25522900
		H	-4.45709400	-0.58764200	2.14745300
		H	-5.04808300	0.36964500	0.80358500
		Se	1.43687200	0.36409600	-0.82660500
		C	3.07550000	-3.79688600	0.61987600
		C	2.77548200	-2.79605400	1.55355400
		H	2.93001800	-2.98306500	2.60134700
		C	2.28614400	-1.57796200	1.13961500
		H	2.06366100	-0.82183300	1.87035400
		C	2.08474900	-1.32735700	-0.21351300
		C	2.38832700	-2.30716300	-1.14429300
		H	2.24782300	-2.12358500	-2.19481300
		C	2.87615400	-3.53552500	-0.73447900
		H	3.10928700	-4.28874300	-1.46562800
		N	3.61481500	-5.00046200	1.03620000
		H	3.41896800	-5.25497400	1.97931000
		H	3.49120100	-5.76583500	0.41043300
		N	-5.02142200	4.31511600	-0.60267800
		O	-6.18800600	4.16114500	-0.28685800
		O	-4.66032400	3.80383100	-1.64764600
		O	2.23060800	1.47997900	0.10907900

**Int 2*** **n_i = 0**	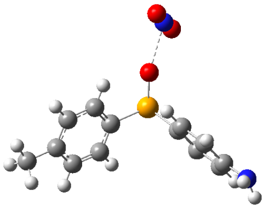	C	-3.95671900	-1.33843100	0.28450200
		C	-3.26747600	-1.68130200	-0.87974900
		H	-3.67147700	-2.43072400	-1.53751200
		C	-2.07505700	-1.06750300	-1.20703400
		H	-1.56507300	-1.34239900	-2.11360300
		C	-1.54861800	-0.10156800	-0.35897100
		C	-2.21653000	0.26347900	0.79200600
		H	-1.81073600	1.02354300	1.43191700
		C	-3.41706600	-0.36063000	1.10808600
		H	-3.93318200	-0.07512100	2.00739900
		C	-5.26054500	-2.01909200	0.62430400
		H	-5.67541100	-1.63235700	1.54702000
		H	-5.11601700	-3.08899700	0.73860600
		H	-5.98956400	-1.86903100	-0.16591300
		Se	0.10221900	0.75945400	-0.83611600
		C	3.26116200	-2.45658000	0.46830600
		C	3.11497000	-2.09427600	-0.87001300
		H	3.73866900	-2.54828900	-1.61889000
		C	2.17689000	-1.14708900	-1.23824400
		H	2.08382000	-0.87689100	-2.27515400
		C	1.36775400	-0.55582900	-0.28161700
		C	1.50922000	-0.90725700	1.05695500
		H	0.89307400	-0.44736100	1.80776300
		C	2.44492200	-1.84419900	1.42957400
		H	2.55308900	-2.11070700	2.46578400
		N	4.23136500	-3.36072800	0.85322300
		H	4.54241900	-3.98847100	0.14499700
		H	4.07117600	-3.82272300	1.72115900
		N	0.95613200	4.19674400	0.43662100
		O	0.65097700	4.22436400	1.47073700
		O	1.28882800	4.25688400	-0.58897600
		O	0.22192700	1.94345600	0.32784300

**TS2**<br><br>**n$_i$ = 1**	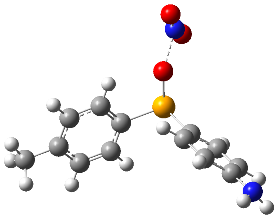	C	-3.76734800	-1.72920500	0.14786800
		C	-3.00552100	-1.92911700	-1.00480700
		H	-3.30324900	-2.67690900	-1.71828900
		C	-1.87568200	-1.17639100	-1.25012600
		H	-1.30752500	-1.34337500	-2.14799600
		C	-1.48955800	-0.21323900	-0.32649300
		C	-2.22810400	0.01267400	0.81678900
		H	-1.92857500	0.76838100	1.51660200
		C	-3.36483000	-0.75180900	1.04658300
		H	-3.93819800	-0.57683100	1.93914300
		C	-5.00247700	-2.56028300	0.39489100
		H	-5.72323900	-2.42138600	-0.40484100
		H	-5.47587500	-2.28950200	1.33047200
		H	-4.75220000	-3.61583600	0.43197400
		Se	0.07160900	0.83472500	-0.70185400
		C	3.62084300	-2.00699800	0.39593000
		C	3.57885700	-1.33858600	-0.82907800
		H	4.37744200	-1.46691400	-1.53689400
		C	2.51785800	-0.50982000	-1.13449500
		H	2.50506300	-0.00099800	-2.08160500
		C	1.48150900	-0.34064600	-0.22773100
		C	1.51253100	-1.00177500	0.99778400
		H	0.71585300	-0.87908400	1.70808700
		C	2.56918700	-1.82273700	1.30749900
		H	2.59176400	-2.33055200	2.25478500
		N	4.69730200	-2.79402900	0.72970400
		H	5.24697400	-3.13614400	-0.02680600
		H	4.54461100	-3.47728500	1.43774600
		N	0.13464400	3.85963300	0.40754400
		O	0.05858000	4.11471200	1.46954400
		O	0.23375200	4.04482200	-0.67148300
		O	0.03211800	1.92512200	0.59112000

**Int 3**<br><br>**n$_i$ = 0**	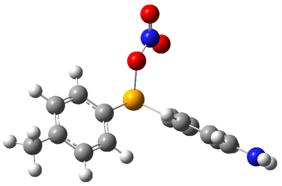	C	-4.03798000	-1.34434500	0.16665500
		C	-2.89906100	-2.10215800	-0.06937800
		H	-2.95708000	-3.17490800	-0.06731300
		C	-1.67490200	-1.50012900	-0.31151600
		H	-0.81382400	-2.10953400	-0.50452400
		C	-1.60119400	-0.11835200	-0.31071400
		C	-2.73158800	0.66896400	-0.11032100
		H	-2.67702500	1.74072500	-0.13688800
		C	-3.93488400	0.04852700	0.13972100
		H	-4.80798800	0.65215400	0.30870200
		C	-5.36397700	-2.00039600	0.45444900
		H	-6.15509000	-1.53733600	-0.12462400
		H	-5.61497800	-1.88969400	1.50526400
		H	-5.33698100	-3.05732800	0.22185400
		Se	0.01509600	0.75402500	-0.81973900
		C	3.60525700	-1.90409500	0.34014300
		C	3.59476000	-1.14037200	-0.84689200
		H	4.44582300	-1.16195900	-1.50128600
		C	2.51164500	-0.36960700	-1.15665200
		H	2.52412600	0.21547300	-2.05834900
		C	1.38889800	-0.35201000	-0.31158300
		C	1.39772200	-1.09715600	0.88351100
		H	0.55977100	-1.06883300	1.55383300
		C	2.48025800	-1.85615600	1.20246300
		H	2.48972500	-2.42464800	2.11361000
		N	4.65955500	-2.66153600	0.65519200
		H	5.45981400	-2.69960600	0.06830800
		H	4.68054200	-3.18758200	1.49723600
		N	0.64787300	3.00202400	0.63962000
		O	0.46302000	3.72023000	1.54369800
		O	1.42608900	3.12270000	-0.24057200
		O	-0.12308500	1.91788600	0.63590900

**TS3*** **n$_i$ = 1**	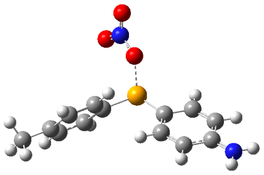	C	-3.49643200	-1.96538100	0.19342600
		C	-3.16629500	-1.65796400	-1.11905300
		H	-3.76692900	-2.03195400	-1.92751500
		C	-2.07004400	-0.86136900	-1.41042900
		H	-1.84086900	-0.62491500	-2.43365100
		C	-1.29057100	-0.38868100	-0.36908800
		C	-1.60538500	-0.66482500	0.95709300
		H	-1.02151300	-0.26410100	1.76282200
		C	-2.70371300	-1.45224000	1.22340900
		H	-2.95380900	-1.66893500	2.24632500
		C	-4.69039200	-2.82672000	0.51802800
		H	-4.38072500	-3.71226100	1.06378200
		H	-5.20414900	-3.14076400	-0.38148900
		H	-5.39170400	-2.28305500	1.14270000
		Se	0.18372700	0.70293100	-0.87649000
		C	4.10936700	-1.23543700	0.37748200
		C	2.87494200	-1.85391200	0.79821900
		H	2.92005500	-2.74657200	1.39206800
		C	1.69870600	-1.32280600	0.44689100
		H	0.79044200	-1.79826100	0.75649800
		C	1.64315200	-0.13945700	-0.37926700
		C	2.88824100	0.47147900	-0.79553600
		H	2.85986300	1.35600800	-1.40484200
		C	4.06812100	-0.05247000	-0.44220700
		H	4.99061900	0.39484400	-0.75929100
		N	5.24900900	-1.75038900	0.73046800
		H	5.29820900	-2.56968900	1.29644900
		H	6.11306700	-1.34134300	0.44813400
		N	-1.02221400	3.01094200	0.68955100
		O	-1.08620900	3.83675800	1.54679700
		O	-1.70638500	3.01924000	-0.29682100
		O	-0.18303500	2.06977500	0.83311800

**Product**<br>$n_i = 0$	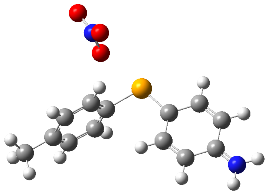	C	2.37005700	-2.94799500	0.00393600
		C	1.90016900	-2.40073800	1.19458900
		H	2.13782200	-2.87175200	2.13127300
		C	1.13132400	-1.25125100	1.19769200
		H	0.78375100	-0.84187500	2.12790200
		C	0.82225800	-0.64614000	-0.01070900
		C	1.29235500	-1.15653300	-1.20960300
		H	1.07000500	-0.67374600	-2.14269300
		C	2.05932300	-2.30824400	-1.19242100
		H	2.42117100	-2.70755300	-2.12259500
		C	3.18259100	-4.21887000	0.01102000
		H	2.52577800	-5.08305800	-0.03763800
		H	3.77094400	-4.29939800	0.91728900
		H	3.85065900	-4.25962700	-0.84081000
		Se	-0.18321400	0.98536700	-0.00524300
		C	-4.58793600	-0.25455900	-0.02318800
		C	-3.56599000	-1.30420300	-0.10249500
		H	-3.88642700	-2.32715300	-0.15429000
		C	-2.27976200	-0.98182400	-0.10531700
		H	-1.52655700	-1.74024500	-0.16215100
		C	-1.86829400	0.41749600	-0.03432300
		C	-2.89357500	1.46364400	0.02148600
		H	-2.57864200	2.48885100	0.06168000
		C	-4.18117300	1.15144500	0.03245500
		H	-4.94374900	1.90488700	0.08079000
		N	-5.82320900	-0.56502000	-0.00665500
		H	-6.12979500	-1.51767000	-0.04505400
		H	-6.54036100	0.13267200	0.04386800
		N	3.08465100	2.53572500	0.01671000
		O	4.14427500	3.13364800	0.01523200
		O	2.56088100	2.21492200	1.07216800
		O	2.53729800	2.25202100	-1.03758300

Table S4. Cartesian coordinates (in Angstrom, Å) of characterized stationary points on the Se oxidation reactions at M06-2X/6-31+G(d,p) level of theory.

**Compound & n_i**	**Structure**	**Atom coordinates**			
**Reactants** **n_i = 0**	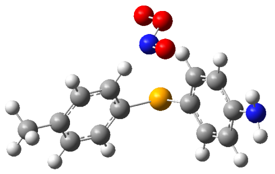	C	-3.69286700	0.85729800	1.02198900
		C	-3.84687100	-0.52988200	0.98331300
		H	-4.70817500	-0.98736200	1.46339200
		C	-2.90499300	-1.34127400	0.34556000
		H	-3.03482300	-2.41942600	0.34042400
		C	-1.79884100	-0.76607900	-0.27555900
		C	-1.63567200	0.62306500	-0.26370900
		H	-0.79263400	1.09031900	-0.77374200
		C	-2.57191900	1.41699500	0.39208900
		H	-2.43872300	2.49689700	0.40240600
		C	-4.69592800	1.73943300	1.72085600
		H	-4.24260000	2.23655900	2.58428000
		H	-5.55266900	1.16121500	2.07380600
		H	-5.06275100	2.52197900	1.05007400
		Se	-0.53517500	-1.88567500	-1.20203500
		C	3.35372700	-0.64793600	1.18550400
		C	2.90291300	0.11975300	0.09972500
		H	3.44369500	1.01905200	-0.18519100
		C	1.76512600	-0.25128500	-0.60809000
		H	1.42291000	0.36780900	-1.43501700
		C	1.04005900	-1.38559500	-0.23388900
		C	1.47850200	-2.14949500	0.85009800
		H	0.92066600	-3.03067700	1.15296100
		C	2.62579900	-1.79261000	1.55027000
		H	2.96020100	-2.39671200	2.38925200
		N	4.52812600	-0.31313200	1.85025800
		H	4.80247200	0.65804400	1.78204900
		H	4.60916200	-0.66368900	2.79547300
		N	1.17708700	3.01219400	-0.18723000
		O	2.08166700	3.38161900	0.52400700
		O	1.59993300	2.76680200	-1.41005900
		O	0.54571300	2.32798000	-2.24158700

**TS1**<br><br>**n_i = 1**	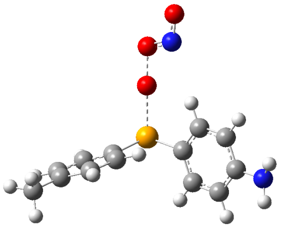	C	-4.04433500	-0.55354200	0.97572100
		C	-4.09622200	-0.02044000	-0.31605000
		H	-5.06131600	0.19869500	-0.76589400
		C	-2.93238500	0.22586600	-1.04302200
		H	-3.00070200	0.62550100	-2.05131000
		C	-1.68565200	-0.04867400	-0.47875100
		C	-1.61530100	-0.56565200	0.81571800
		H	-0.64931200	-0.77602300	1.26591000
		C	-2.78497200	-0.81985100	1.52689500
		H	-2.71821000	-1.22640800	2.53325100
		C	-5.30362500	-0.85380100	1.74838600
		H	-5.54188400	-1.92189300	1.70472000
		H	-6.15625700	-0.30530600	1.34137500
		H	-5.19215000	-0.58574900	2.80243000
		Se	-0.12350400	0.37091500	-1.49661600
		C	2.97488600	-2.56081600	0.51674100
		C	3.00800900	-1.18279300	0.78517000
		H	3.76193600	-0.79046900	1.46259700
		C	2.08775800	-0.32110500	0.19552100
		H	2.09745000	0.74577000	0.40572200
		C	1.12011100	-0.82236700	-0.67480900
		C	1.08243800	-2.19038700	-0.95489300
		H	0.33062500	-2.58582400	-1.63231500
		C	1.99412800	-3.05455900	-0.36233200
		H	1.95873200	-4.11789000	-0.58285800
		N	3.93013700	-3.41167200	1.05762400
		H	4.37845700	-3.08576700	1.90343600
		H	3.66767900	-4.38647100	1.11557200
		N	2.15329100	3.37037100	0.98237700
		O	2.63694700	4.33229200	1.57138800
		O	1.12873600	3.66040800	0.28523600
		O	0.56458800	2.25179500	-0.48475100

**Int 1** **n$_i$ = 0**	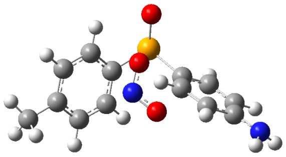	C	-3.57816700	0.39333900	1.00689500
		C	-2.54965700	0.09225500	1.91302100
		H	-2.62981800	0.42823600	2.94348600
		C	-1.42588800	-0.61759800	1.50742600
		H	-0.62846700	-0.83090900	2.21479900
		C	-1.33165000	-1.03871700	0.18245600
		C	-2.33931800	-0.77364200	-0.73220400
		H	-2.23199000	-1.11061900	-1.75907600
		C	-3.45878300	-0.05131100	-0.31229800
		H	-4.24813700	0.17236100	-1.02480000
		C	-4.77566900	1.19209500	1.45287000
		H	-4.50244100	2.24146600	1.60401600
		H	-5.16778800	0.81554800	2.40150900
		H	-5.57393200	1.15531600	0.70868400
		Se	0.18890000	-2.07380200	-0.38773100
		C	3.63410500	0.94876100	0.67210700
		C	2.42350800	1.42349400	0.12861000
		H	2.28541900	2.48212100	-0.07266000
		C	1.39069300	0.54566900	-0.15299900
		H	0.47072600	0.93843500	-0.58063400
		C	1.54181900	-0.81953900	0.10058800
		C	2.73653200	-1.30258600	0.63432700
		H	2.86099300	-2.36346200	0.83502300
		C	3.77490700	-0.42678400	0.92670800
		H	4.70236200	-0.80262700	1.34832800
		N	4.68169400	1.81761900	0.90472300
		H	4.44690500	2.79585600	0.99928200
		H	5.39202300	1.51227500	1.55530900
		N	-0.91205400	3.05128800	-1.20073500
		O	0.11002800	3.54304700	-0.67717000
		O	-0.72623800	2.38876600	-2.24357500
		O	0.08614600	-1.98177400	-2.06467700

**Int 2***<br><br>**n$_i$ = 0**	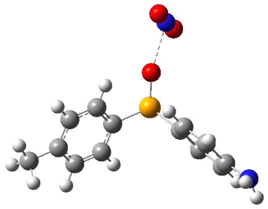	*This structure could not be optimized at B3LYP/6-311+G(d,p) level of theory. The reported structure was fully optimized at HF/6-311+G(d,p) level of theory.
**TS2**<br><br>**n$_i$ = 1**	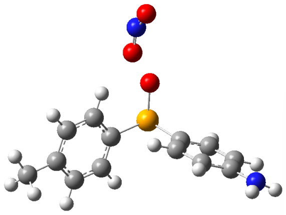	C -3.36324200 -2.25864500 0.24970000<br>C -2.54336700 -2.48625800 -0.86608200<br>H -2.70567900 -3.36890900 -1.47829600<br>C -1.52918400 -1.59875300 -1.20270800<br>H -0.90167500 -1.78823600 -2.06941000<br>C -1.33398100 -0.47627800 -0.40028300<br>C -2.13306400 -0.20820800 0.70131900<br>H -1.96480100 0.68569700 1.29291000<br>C -3.14712300 -1.11269700 1.02006700<br>H -3.77837700 -0.91975900 1.88251600<br>C -4.44754400 -3.24150800 0.60481000<br>H -5.07060800 -2.86592000 1.41855700<br>H -4.01184600 -4.19467900 0.91965700<br>H -5.08784900 -3.44306500 -0.25834100<br>Se 0.03777100 0.75617800 -0.90741300<br>C 3.91770700 -1.41748100 0.53003700<br>C 3.93870700 -0.53270300 -0.57136100<br>H 4.87375200 -0.34384500 -1.08881900<br>C 2.77662400 0.08665100 -0.99245600<br>H 2.80590800 0.76194500 -1.84249500<br>C 1.57310400 -0.17038800 -0.32661500<br>C 1.53233100 -1.04374900 0.76580600<br>H 0.59583300 -1.24427700 1.27799800<br>C 2.69166300 -1.65932900 1.19292000<br>H 2.66633400 -2.33966400 2.03817000<br>N 5.06384400 -2.00740300 0.96621100<br>H 5.89224400 -1.97515700 0.39161500<br>H 5.01378600 -2.76556100 1.62973000<br>N -1.07622100 4.26879900 0.47201900<br>O -0.71908100 4.49434800 1.54895600<br>O -1.07423900 3.46032400 -0.35571900<br>O -0.09673000 1.91753700 0.36172800

**Int 3**<br><br>**n$_i$ = 0**	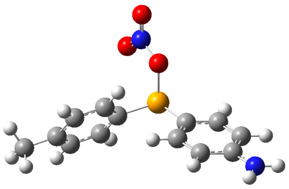	C -3.48895600 -1.94506900 0.14756000<br>C -3.15400700 -1.59578600 -1.16460200<br>H -3.75024500 -1.97020600 -1.99067700<br>C -2.07275400 -0.76068200 -1.42983300<br>H -1.83404300 -0.48240700 -2.45166900<br>C -1.31961200 -0.29551300 -0.35550100<br>C -1.63139200 -0.60536500 0.96862500<br>H -1.04940000 -0.20285200 1.79193900<br>C -2.72061400 -1.43246700 1.20503800<br>H -2.98243000 -1.68481200 2.22823300<br>C -4.65044900 -2.85631100 0.43476100<br>H -5.27050400 -2.45349900 1.23945300<br>H -4.28792300 -3.83731100 0.75785100<br>H -5.27202600 -2.99756000 -0.45084600<br>Se 0.12294000 0.83604100 -0.81184400<br>C 4.07133500 -1.30064100 0.31226600<br>C 4.04554900 -0.14663400 -0.51525200<br>H 4.97521800 0.25277100 -0.90574800<br>C 2.85070500 0.45377300 -0.82521900<br>H 2.84256200 1.33168100 -1.46438300<br>C 1.64438100 -0.07023900 -0.31175800<br>C 1.65266900 -1.22098600 0.50104200<br>H 0.72772100 -1.64685800 0.87293600<br>C 2.84545100 -1.82728000 0.80562800<br>H 2.85962300 -2.72022700 1.42120900<br>N 5.23558500 -1.89485300 0.61657100<br>H 6.11185000 -1.53745200 0.26695400<br>H 5.26764000 -2.71650700 1.20103000<br>N -0.95798500 2.87277300 0.70650700<br>O -0.97954300 3.56310600 1.68301300<br>O -1.68253000 2.88654300 -0.25968700<br>O 0.05019200 1.92705200 0.72521500
**TS3***<br><br>**n$_i$ = 1**	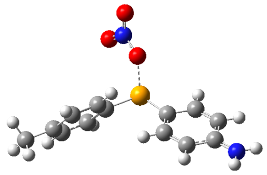	This structure could not be optimized at B3LYP/6-311+G(d,p) level of theory. The reported structure was fully optimized at HF/6-311+G(d,p) level of theory.

**Product**<br><br>**n$_i$ = 0**	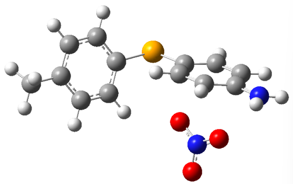	C	4.12475500	0.91419200	0.59157300
		C	4.09275300	-0.47754700	0.75493000
		H	4.92832200	-0.98006100	1.23344400
		C	3.00757800	-1.22818800	0.31669300
		H	2.99484500	-2.30466100	0.45227000
		C	1.94010400	-0.56584300	-0.28845600
		C	1.94518600	0.81589500	-0.48350000
		H	1.10625800	1.30912600	-0.96677100
		C	3.04123300	1.54509900	-0.03131200
		H	3.05539200	2.62189100	-0.17102200
		C	5.29778700	1.71086200	1.09582500
		H	5.30930400	2.71399400	0.66588300
		H	6.24059600	1.21416900	0.85378600
		H	5.24731600	1.80894200	2.18501500
		Se	0.49420800	-1.59244500	-0.97721700
		C	-3.11406900	-0.25842000	1.50333800
		C	-1.78073400	0.14646100	1.93861400
		H	-1.68752800	0.76404100	2.82494000
		C	-0.70503300	-0.24670400	1.23649800
		H	0.29223000	0.04579800	1.54203900
		C	-0.87167700	-1.06855000	0.05805100
		C	-2.18664300	-1.56198500	-0.31219300
		H	-2.28078800	-2.22188800	-1.16772700
		C	-3.27223300	-1.16758500	0.37313200
		H	-4.26875800	-1.49709200	0.10017600
		N	-4.15347300	0.14889200	2.15843000
		H	-4.06822800	0.77684100	2.95370700
		H	-5.09614800	-0.11892600	1.88723700
		N	-2.21568100	1.86770800	-1.12762100
		O	-2.31649600	2.85923500	-1.85604700
		O	-3.01871400	1.68364400	-0.18500900
		O	-1.30301800	1.02569800	-1.32174800

Table S5. Electronic energies (EE), relative energies (ΔE), and the activation energies (E_a) for stationary points anchored on the potential energy surface at M06-2X/6-31+G(d,p) level of theory.

Step #	Compound	M06-2X/6-31+G(d,p)		
		**EE[a]**	**ΔE[b]**	**E_a [c]**
	Reactants	-3237.507890	0.00	N/A
**1**	TS1	-3237.482565	66.49	66.49
	Intermediate1	-3237.575566	-177.68	N/A
**2**		2e⁻ transfer[d]		
	Intermediate2[e]	**-3237.142590**	**0.00**	**N/A**
**3**	TS2	-3237.126578	42.04	42.04
	Intermediate3	-3237.209875	-176.66	N/A
**4**	TS3[e]	**-3237.189938**	**-166.35**	**10.31**
	Products	-3237.188139	-119.59	N/A

[a] Electronic Energy in Hartree.
[b] In kJ/mol, for stationary points anchored in step 1, ΔE (energy relative to Intermediate2) was calculated using the equation $\Delta E = (E_{target\ molecule} - E_{reactants}) \times 2625.5$, otherwise, it is determined using equation $\Delta E = (E_{target\ molecule} - E_{intermediate\ 2}) \times 2625.5$.
[c] Activation energy, in kJ/mol.
[d] The Cartesian Coordinates are listed in **Table S4**.
[e] Structure vanishes when the DFT method is applied due to nature of the PES (**Table S1, Figure S1**). Single point energetics (in green) at M06-2X/6-31+G(d,p) level of DFT theory based on structures obtained at HF/6-311+G(d,p) level of *ab initio* theory.

Table S6. Single point energy extrapolated at different levels of theory to estimate the thermodynamic aspect of Step 1 and Step 3.

Structure	HF			B3LYP
	**6-31G(d,p)**	**6-31+G(d,p)**	**6-311+G(d,p)**	**6-311+G(d,p)**
$EE_{Int1\&HOOH}$[a]	-3381.478724	-3381.638378	-3384.007656	-3391.683242
$EE_{Int2\&OH^-}$[a]	-3381.363558	-3381.485189	-3383.855248	-3391.54158
EE_{gap}[a,b]	0.115166	0.153189	0.152408	0.141662
E_{gap}[c]	302.37	402.20	400.15	371.93

[a] Electronic Energy in Hartree. [b] Determined using equation $EE_{gap} = EE_{Int2\&OH^-} - EE_{Int1\&HOOH}$
[c] In kJ/mol, determined using equation $E_{gap} = EE_{gap} \times 2625.5$

Table S7. Cartesian coordinates (in Angstrom, Å) of the two proposed stationary points (based on Int 1 and Int2) on PES.

**Compound & n_i**	**Structure**	**Atom coordinates**			
**Int1&HOOH Proposed Stationary point**	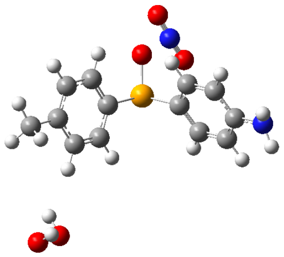	C	-0.64411800	4.29666600	0.30900400
		C	-0.03422000	3.59251000	-0.73080700
		H	0.33203200	4.12435400	-1.59152000
		C	0.11064600	2.22168300	-0.66994900
		H	0.58710700	1.69663300	-1.47942200
		C	-0.36386400	1.53536700	0.43967600
		C	-0.96412900	2.20795600	1.48363300
		H	-1.31378700	1.66521100	2.34081500
		C	-1.10122500	3.58787600	1.41109200
		H	-1.56985200	4.11148200	2.22552900
		C	-0.79708000	5.79624200	0.22325900
		H	-1.21681300	6.20103700	1.13578800
		H	-1.45111200	6.06965800	-0.59909700
		H	0.16170700	6.27391900	0.04962700
		Se	-0.15081600	-0.36306400	0.51844900
		C	-3.63795900	-1.66085800	-2.31493300
		C	-2.30803100	-1.84896300	-2.69590000
		H	-2.08281400	-2.30321200	-3.64416800
		C	-1.28153600	-1.46353500	-1.85519100
		H	-0.26179400	-1.62212300	-2.15846100
		C	-1.56311800	-0.87630800	-0.63300300
		C	-2.88400700	-0.69097500	-0.24018200
		H	-3.10035600	-0.25011900	0.71540800
		C	-3.91095200	-1.07746300	-1.06868200
		H	-4.93205400	-0.93559600	-0.76193900
		N	-4.66899800	-2.09225400	-3.12087600
		H	-4.45307200	-2.19317700	-4.08787400
		H	-5.55037700	-1.65273200	-2.97304600
		N	0.42420400	-3.16750400	2.75148200
		O	0.05820000	-2.81586100	3.71540500
		O	0.76779900	-3.64073100	1.82932700
		O	-0.76208700	-0.71613000	2.02925700
		O	9.60061700	0.23648300	-1.61464800
		H	8.64069200	0.15908000	-1.71008600
		O	9.98108400	-1.15305300	-1.45282800
		H	10.16446000	-1.18881000	-0.50327500

**Int2&2OH⁻** **Proposed Stationary point**	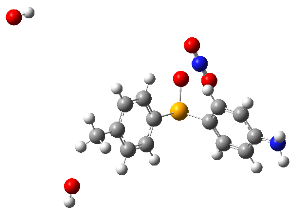	C	-0.25795000	4.17621300	-0.03607600
		C	-0.08450800	3.48773600	1.16597900
		H	-0.04203300	4.03444100	2.09179400
		C	0.02924800	2.11277500	1.18593500
		H	0.15840100	1.59921400	2.12269200
		C	-0.02343800	1.40770200	-0.00886700
		C	-0.20092300	2.06319600	-1.20943700
		H	-0.25321700	1.50338900	-2.12348000
		C	-0.31678900	3.44695100	-1.21533100
		H	-0.45476000	3.95729700	-2.15213000
		C	-0.37694800	5.68130800	-0.03874200
		H	-0.58372700	6.05717900	-1.03308000
		H	0.54274500	6.14037300	0.31084000
		H	-1.17494200	6.00775900	0.62021900
		Se	0.12666100	-0.49797100	0.03004400
		C	4.75412100	-0.90837400	0.56918900
		C	3.88540800	-1.27610600	1.59843100
		H	4.28195800	-1.66457100	2.51936600
		C	2.51910400	-1.15262400	1.43410100
		H	1.86162000	-1.44755800	2.23267800
		C	2.00044700	-0.65166300	0.25177300
		C	2.85599800	-0.28933700	-0.78271100
		H	2.45085700	0.08378400	-1.70518300
		C	4.21650200	-0.41500200	-0.62896200
		H	4.87654400	-0.13659200	-1.43127100
		N	6.11494700	-1.07966400	0.70147600
		H	6.46047900	-1.11809900	1.63476400
		H	6.68428400	-0.51663600	0.10926200
		N	-0.97366300	-3.56760700	-1.56638800
		O	-1.24159900	-3.25794300	-2.57588200
		O	-0.68536600	-3.99698500	-0.60467600
		O	-0.08671000	-0.89340500	-1.57586800
		O	-7.84922800	-1.74420000	5.93191800
		H	-8.09912000	-1.85588900	6.85206900
		O	-10.26055700	0.08751500	-3.11771700
		H	-10.01849200	-0.83868500	-3.18954400

Figure S2. A rough estimation of the potential energy surface near the oxygen-transfer transition state for the reaction converting NO_2^- and $ONOO^-$ to NO_2^- and NO_3^-

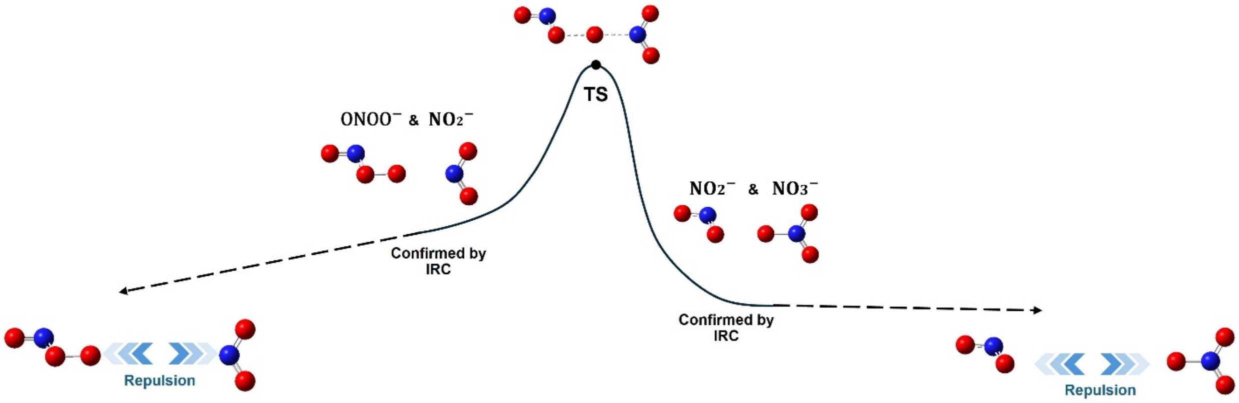

Table S8. Cartesian coordinates (in Angstrom, Å) of the oxygen transfer TS to convert NO_2^- and $ONOO^-$ to NO_2^- and NO_3^- calculated at HF/6-31G(d,p) level of theory.

**Compound & n$_i$**	**Structure**	**Atom coordinates**			
**TS**<br><br>**n$_i$ = 1**	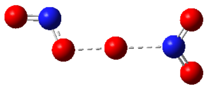	N<br>O<br>O<br>N<br>O<br>O<br>O	2.12566300<br>2.88837900<br>2.61329400<br>-2.39335700<br>-3.60112900<br>-1.75207900<br>0.08576700	0.04603500<br>-0.88297600<br>1.15973400<br>0.34456700<br>0.23348300<br>-0.58192500<br>-0.27009200	-0.01325000<br>-0.22835600<br>0.10096800<br>-0.20416200<br>-0.14613900<br>0.31190200<br>0.15186100

Table S9. Cartesian coordinates (in Angstrom, Å) of the oxygen transfer TS to convert NO_2^- and $ONOO^-$ to NO_2^- and NO_3^- calculated at HF/6-31+G(d,p) level of theory.

**Compound & n$_i$**	**Structure**	**Atom coordinates**			
**TS**<br><br>**n$_i$ = 1**	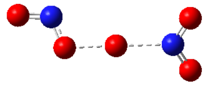	N<br>O<br>O<br>N<br>O<br>O<br>O	2.12282700<br>2.88368300<br>2.60465100<br>-2.39364300<br>-3.59990200<br>-1.73895200<br>0.08748500	0.04592900<br>-0.88469200<br>1.16107700<br>0.34394200<br>0.22705100<br>-0.57668200<br>-0.26789000	-0.01275000<br>-0.21842600<br>0.09520700<br>-0.19729900<br>-0.13932100<br>0.30017400<br>0.14615900

Table S10. Cartesian coordinates (in Angstrom, Å) of the oxygen transfer TS to convert NO_2^- and $ONOO^-$ to NO_2^- and NO_3^- calculated at HF/6-311+G(d,p) level of theory.

Compound & n_i	Structure	Atom coordinates			
**TS** n_i = 1	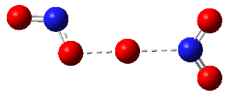	N	2.13416100	0.04485200	-0.01337800
		O	2.88612100	-0.88441600	-0.22182000
		O	2.61674500	1.15296900	0.09498600
		N	-2.40466300	0.33931200	-0.20445500
		O	-3.60375200	0.22036400	-0.14216000
		O	-1.74614700	-0.56228200	0.30841000
		O	0.08372300	-0.26277800	0.15118900

Table S11. Cartesian coordinates (in Angstrom, Å) of the oxygen transfer TS to convert NO_2^- and $ONOO^-$ to NO_2^- and NO_3^- calculated at B3LYP/6-311+G(d,p) level of theory.

Compound & n_i	Structure	Atom coordinates			
**TS** n_i = 1	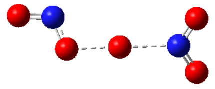	N	2.09612900	0.04325000	-0.01819800
		O	2.86796600	-0.91516500	-0.22522800
		O	2.59614900	1.18035500	0.09898200
		N	-2.36557600	0.36429700	-0.23309100
		O	-3.58414300	0.19096400	-0.13558600
		O	-1.66849200	-0.55482100	0.32950300
		O	0.02428500	-0.25793700	0.15220600

Table S12. The electronic energy (in Hartree) determined for single-point energy calculations performed on the proposed structures to convert NO_2^- and $ONOO^-$ to NO_2^- and NO_3^-

Structure[a]	HF			B3LYP
	6-31G(d,p)	6-31+G(d,p)	6-311+G(d,p)	6-311+G(d,p)
**R60**	-483.086784	-483.142475	-483.267069	-485.785906
**R50**	-483.086761	-483.142452	-483.267046	-485.785883
**R40**	-483.086728	-483.142418	-483.267012	-485.785848
**R30**	-483.086670	-483.142360	-483.266954	-485.785789
**R20**	-483.086552	-483.142240	-483.266834	-485.785666
**R10**	-483.086165	-483.141845	-483.266439	-485.785257
**TS**	-483.051706	-483.104660	-483.229894	-485.766052
**P10**	-483.185587	-483.237940	-483.363592	-485.873538
**P20**	-483.185947	-483.238302	-483.363954	-485.873902
**P30**	-483.186064	-483.238419	-483.364072	-485.874020
**P40**	-483.186116	-483.238471	-483.364124	-485.874073
**P50**	-483.186149	-483.238505	-483.364158	-485.874107
**P60**	-483.186171	-483.238527	-483.364180	-485.874130

[a]The number X in PX and RX indicate that the two nitrogen atoms in the proposed structures are X Angstrom (Å) apart when the single-point energy was calculated.

Table S13. The relative energy (in kJ/mol) determined for single-point energy calculations performed on the proposed structures to convert NO_2^- and $ONOO^-$ to NO_2^- and NO_3^-

Structure[a]	HF			B3LYP
	6-31G(d,p)	6-31+G(d,p)	6-311+G(d,p)	6-311+G(d,p)
**R60**	0.00	0.00	0.00	0.00
**R50**	0.06	0.06	0.06	0.06
**R40**	0.15	0.15	0.15	0.15
**R30**	0.30	0.30	0.30	0.31
**R20**	0.61	0.62	0.62	0.63
**R10**	1.63	1.65	1.65	1.70
**TS**	92.10	99.28	97.60	52.13
**P10**	-259.41	-250.64	-253.42	-230.08
**P20**	-260.35	-251.59	-254.37	-231.03
**P30**	-260.66	-251.90	-254.68	-231.34
**P40**	-260.80	-252.04	-254.82	-231.48
**P50**	-260.88	-252.13	-254.91	-231.57
**P60**	-260.94	-252.18	-254.96	-231.63

[a]The number X in PX and RX indicate that the two nitrogen atoms in the proposed structures are X Angstrom (Å) apart when the single-point energy was determined.

Figure S3. A rough estimation of the potential energy surface near the oxygen-transfer transition state for the reaction converting NO_2^- and $ONOO^-$ to NO_2^- and NO_3^- based on single-point energy calculations performed on the proposed structures

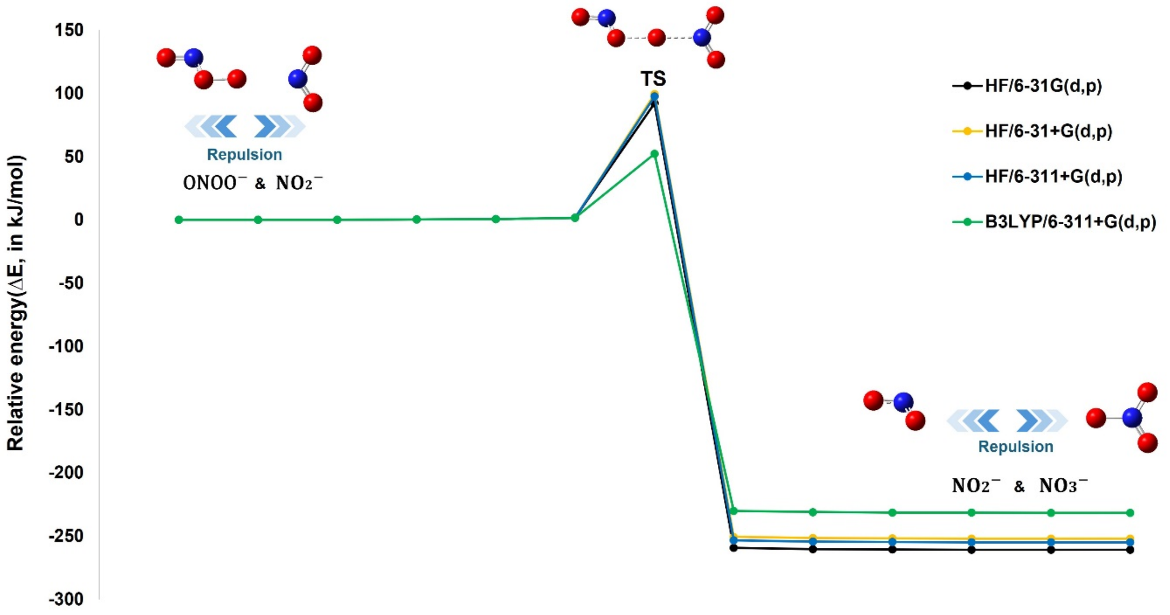

Additional notes:

This figure is constructed based on values from **Table S13**.

Table S14. The electronic energy (in Hartree) determined by constrained geometry optimization with frozen N…N distances to convert NO_2^- and $ONOO^-$ to NO_2^- and NO_3^-

**Structure**[a]	**HF**			**B3LYP**
	**6-31G(d,p)**	**6-31+G(d,p)**	**6-311+G(d,p)**	**6-311+G(d,p)**
**R60**	-483.086805	-483.142612	-483.267809	-485.792531
**R50**	-483.086782	-483.142589	-483.267785	-485.792507
**R40**	-483.086748	-483.142554	-483.267750	-485.792472
**R30**	-483.086689	-483.142494	-483.267690	-485.792411
**R20**	-483.086569	-483.142370	-483.267568	-485.792295
**R10**	-483.086171	-483.141969	-483.267163	-485.791870
**TS**	-483.051706	-483.104660	-483.229894	-485.766052
**P10**	-483.186122	-483.238264	-483.363652	-485.883050
**P20**	-483.186455	-483.238604	-483.364013	-485.883403
**P30**	-483.186558	-483.238738	-483.364127	-485.883517
**P40**	-483.186618	-483.238777	-483.364166	-485.883573
**P50**	-483.186652	-483.238800	-483.364207	-485.883621
**P60**	-483.186699	-483.238843	-483.364229	-485.883643

[a]The number X in PX and RX indicate that the two nitrogen atoms in the proposed structures are X Angstrom (Å) apart when the constrained geometry optimization was conducted.

Table S15. The relative energy (in kJ/mol) determined by constrained geometry optimization (with frozen N…N distances) to convert NO_2^- and $ONOO^-$ to NO_2^- and NO_3^-

**Structure**[a]	**HF**			**B3LYP**
	**6-31G(d,p)**	**6-31+G(d,p)**	**6-311+G(d,p)**	**6-311+G(d,p)**
**R60**	0.00	0.00	0.00	0.00
**R50**	0.06	0.06	0.06	0.06
**R40**	0.15	0.15	0.15	0.15
**R30**	0.30	0.31	0.31	0.32
**R20**	0.62	0.64	0.63	0.62
**R10**	1.66	1.69	1.70	1.74
**TS**	92.15	99.64	99.55	69.52
**P10**	-260.76	-251.13	-251.64	-237.66
**P20**	-261.63	-252.03	-252.58	-238.58
**P30**	-261.90	-252.38	-252.88	-238.88
**P40**	-262.06	-252.48	-252.99	-239.03
**P50**	-262.15	-252.54	-253.09	-239.16
**P60**	-262.27	-252.65	-253.15	-239.21

[a]The number X in PX and RX indicate that the two nitrogen atoms in the proposed structures are X Angstrom (Å) apart when the constrained geometry optimization was conducted.

Figure S4. The potential energy surface for the reaction converting NO_2^- and $ONOO^-$ to NO_2^- and NO_3^- based on constrained geometry optimization performed on the proposed structures

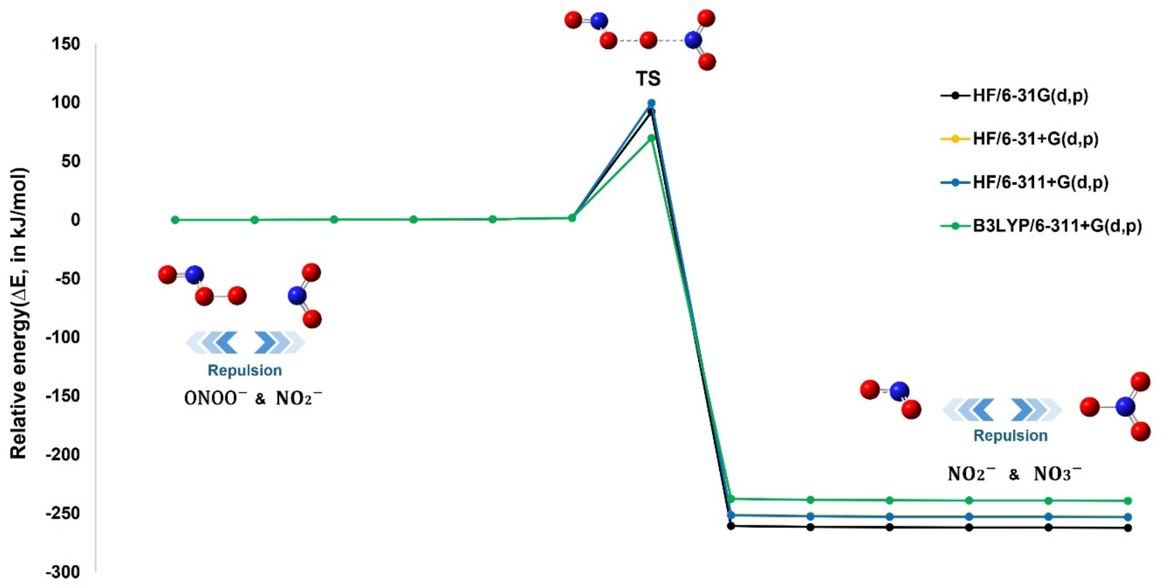

Additional notes:

This figure is constructed based on values from **Table S15**.

Table S16. A summary of E_a and ΔH (in kJ/mol) for the reaction of converting NO_2^- and $ONOO^-$ to NO_2^- and NO_3^-

Quantity	Computation Method	HF			B3LYP
		6-31G(d,p)	6-31+G(d,p)	6-311+G(d,p)	6-311+G(d,p)
E_a	SP[1]	92.10	99.28	97.60	52.13
	Constr. Geom. [2]	92.15	99.64	99.55	69.52
ΔH	SP[3]	-260.94	-252.18	-254.96	-231.63
	Constr. Geom. [4]	-262.27	-252.65	-253.15	-239.21

[1]Table S13;

[2]Table S15;

[3]Table S13;

[4]Table S15.

Table S17. Cartesian coordinates (in Angstrom, Å) of characterized stationary points on PES (Depicted in Scheme 3 in manuscript) calculated at HF/6-31G(d,p) level of theory

**Compound & n_i**	**Structure**	**Atom coordinates**			
**Reactants'** $n_i = 0$	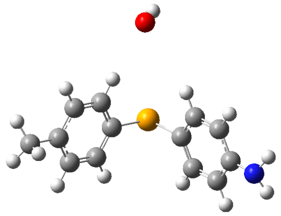	C	1.46081000	-2.66916200	-0.71563600
		C	1.29647700	-1.94730200	-1.89377100
		H	1.48085200	-2.42490800	-2.84038300
		C	0.90300700	-0.61941100	-1.86940600
		H	0.78708700	-0.06092000	-2.77901100
		C	0.66764900	-0.00272500	-0.65438300
		C	0.84101400	-0.69642800	0.53181000
		H	0.67883000	-0.21044900	1.47818600
		C	1.23103300	-2.02372100	0.49660700
		H	1.36545100	-2.55908100	1.42022400
		C	1.86207400	-4.12459600	-0.75027500
		H	0.98303500	-4.76275200	-0.77826100
		H	2.43590700	-4.39433900	0.12869800
		H	2.45760900	-4.34628300	-1.62809400
		Se	0.13797000	1.83700200	-0.61224500
		C	-4.50697700	1.22061500	-0.40216400
		C	-3.83427600	1.17611000	-1.62852800
		H	-4.39007500	1.00136300	-2.53298400
		C	-2.46878700	1.35535800	-1.68361100
		H	-1.95710800	1.33419200	-2.62782100
		C	-1.74685800	1.57871600	-0.52030400
		C	-2.40411800	1.63965900	0.69744200
		H	-1.85529200	1.83282100	1.60275800
		C	-3.77237900	1.45718600	0.76129000
		H	-4.27641000	1.50399500	1.71041400
		N	-5.86896200	0.98731000	-0.34086600
		H	-6.37279300	1.17955600	-1.17868900
		H	-6.32938100	1.35845600	0.46094700
		O	1.32548500	2.76156100	-4.31832100
		H	1.59061900	3.66241600	-4.47161500

**Intermediate1'**<br><br>**n$_i$ = 0**	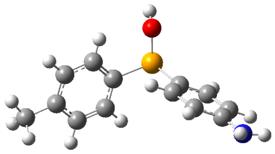	C	1.38567300	-2.81249400	-0.44580600
		C	1.46368300	-1.90938300	-1.49651600
		H	1.87343500	-2.22006500	-2.44042700
		C	1.02059200	-0.60178900	-1.35542700
		H	1.08929700	0.08489100	-2.17624200
		C	0.49233100	-0.20992400	-0.14260600
		C	0.40824200	-1.08706200	0.93172500
		H	-0.00089800	-0.77073500	1.87500000
		C	0.85173000	-2.38176200	0.77080900
		H	0.78588400	-3.06626200	1.59750000
		C	1.86367900	-4.23521400	-0.60055300
		H	1.04179100	-4.93101700	-0.46544400
		H	2.61708300	-4.47022100	0.14396500
		H	2.29084300	-4.40192900	-1.58116700
		Se	-0.09873700	1.57328300	0.15891800
		C	-4.73053000	1.24824700	0.16862300
		C	-3.97717000	0.47011500	-0.73137300
		H	-4.48287900	-0.18796600	-1.41405700
		C	-2.60997800	0.54352200	-0.73825400
		H	-2.05374400	-0.06187900	-1.42911400
		C	-1.95398600	1.39866200	0.14945000
		C	-2.68753400	2.17704800	1.04141300
		H	-2.18798200	2.83995400	1.72454200
		C	-4.05990400	2.10237500	1.05588100
		H	-4.62283400	2.69984800	1.74908700
		N	-6.08446500	1.13745000	0.20294500

**TS1’**<br><br>**n$_i$ = 0**	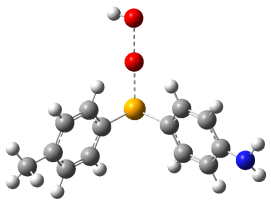	C	1.69856500	-2.85996500	-0.28299000
		C	0.50255700	-2.85608500	-1.00449200
		H	0.07879500	-3.78841800	-1.33522500
		C	-0.14485600	-1.67635700	-1.30496500
		H	-1.06136500	-1.69359200	-1.86458500
		C	0.39584400	-0.46793700	-0.87936300
		C	1.57784900	-0.45132900	-0.16275900
		H	1.99693700	0.48026700	0.17156800
		C	2.22464700	-1.64514500	0.12938000
		H	3.14362300	-1.62026700	0.68757400
		C	2.38841900	-4.16418200	0.03627600
		H	2.60318700	-4.71958500	-0.87110900
		H	1.75811000	-4.79062400	0.65990000
		H	3.32157200	-3.99780600	0.55999500
		Se	-0.44725000	1.18484000	-1.29856800
		C	-4.78956200	0.48989800	0.33442800
		C	-4.54186300	0.43502400	-1.03823500
		H	-5.35178900	0.23831500	-1.71825400
		C	-3.26557700	0.62520500	-1.53275700
		H	-3.09334000	0.57737600	-2.59070100
		C	-2.21317300	0.87626300	-0.66539200
		C	-2.45161100	0.94255800	0.70261400
		H	-1.64263200	1.14479000	1.38145200
		C	-3.72114700	0.74737900	1.20045500
		H	-3.89399600	0.79574800	2.26092500
		N	-6.05377800	0.24485200	0.83441800
		H	-6.80592500	0.41228900	0.20298300
		H	-6.23680600	0.61819400	1.73968200
		O	-0.75928600	0.71708600	-3.32796400
		H	-0.58458600	1.56406700	-3.71741500
		O	-0.98264100	0.50259700	-5.20428200
		H	-0.28552000	-0.12615000	-5.31470200

**Products1'**<br>**n$_i$ = 0**	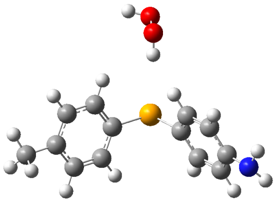	C	1.60710300	-2.96617900	-0.32646200
		C	1.09156000	-2.74083800	-1.59468100
		H	1.16480200	-3.50829700	-2.34538100
		C	0.47598300	-1.53774500	-1.91490800
		H	0.08484300	-1.39084900	-2.90488400
		C	0.37202300	-0.53495600	-0.96621900
		C	0.89471800	-0.74582800	0.30534100
		H	0.82767700	0.02979700	1.04638200
		C	1.49855300	-1.94741900	0.61986500
		H	1.89613900	-2.09532700	1.60937200
		C	2.26509500	-4.27697800	0.03341000
		H	2.38588600	-4.90866600	-0.83820300
		H	1.66986900	-4.82032100	0.76145100
		H	3.24429900	-4.11312000	0.47149400
		Se	-0.44928900	1.14117700	-1.39784400
		C	-4.64840500	0.56805600	0.66610000
		C	-4.33974200	-0.11263400	-0.51460800
		H	-5.06942700	-0.76830200	-0.95694500
		C	-3.10996600	0.05270300	-1.11847000
		H	-2.90165500	-0.47847300	-2.02933000
		C	-2.14884300	0.88959200	-0.56314200
		C	-2.45425000	1.56402600	0.61048900
		H	-1.72518800	2.21629900	1.05582800
		C	-3.68635000	1.41175000	1.21972800
		H	-3.90261400	1.94383000	2.12960400
		N	-5.86507200	0.36587800	1.29985100
		H	-6.60508700	0.06627400	0.70333000
		H	-6.15705200	1.10897400	1.89641000
		O	-1.37953100	0.49221000	-4.76447400
		H	-1.13878700	0.70464900	-3.86827400
		O	-0.63395900	1.40073800	-5.51566100
		H	0.10007100	0.88288400	-5.81647800

**Intermediate2'**<br><br>**n$_i$ = 0**	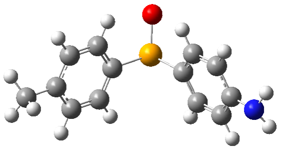	C	1.30477300	-2.79015600	-0.22857900
		C	1.13574000	-2.06982300	-1.40704600
		H	1.31611900	-2.54877300	-2.35375100
		C	0.74251100	-0.74194200	-1.38276400
		H	0.62277300	-0.18467800	-2.29260800
		C	0.51225500	-0.12353000	-0.16760500
		C	0.69018800	-0.81567200	1.01878600
		H	0.53178600	-0.32850500	1.96519200
		C	1.07990700	-2.14311400	0.98369100
		H	1.21788600	-2.67729700	1.90744800
		C	1.70571500	-4.24566400	-0.26303200
		H	0.82645900	-4.88368000	-0.28651200
		H	2.28318500	-4.51436400	0.61385800
		H	2.29750100	-4.46853100	-1.14308200
		Se	-0.01734700	1.71613400	-0.12603900
		C	-4.66233800	1.09927500	0.08137700
		C	-3.98970600	1.06007900	-1.14522700
		H	-4.54551300	0.88878200	-2.05034000
		C	-2.62420700	1.23963100	-1.19963600
		H	-2.11252000	1.22196200	-2.14391600
		C	-1.90225300	1.45836600	-0.03551800
		C	-2.55944200	1.51417100	1.18257900
		H	-2.01058400	1.70340600	2.08869800
		C	-3.92759500	1.33125900	1.24576500
		H	-4.43150900	1.37390600	2.19513700
		N	-6.02402100	0.86526900	0.14192700
		H	-6.52847200	1.05998200	-0.69491600
		H	-6.48469400	1.23167100	0.94573700
		O	0.23195200	2.21555700	-1.68323600

**TS2'** **n_i = 1**	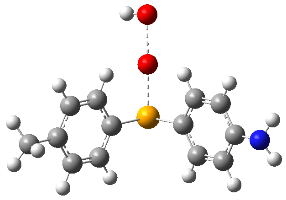	C	1.35875900	-2.77450000	-0.24926400
		C	1.15410300	-2.06116200	-1.42604600
		H	1.30658000	-2.54698400	-2.37468300
		C	0.76190600	-0.73211300	-1.40012600
		H	0.61893300	-0.17973600	-2.30944900
		C	0.56418600	-0.09396600	-0.18536500
		C	0.77650900	-0.78909400	0.99621000
		H	0.63929300	-0.29580400	1.94166800
		C	1.16782000	-2.11684700	0.96211100
		H	1.33028900	-2.64335500	1.88691200
		C	1.75932500	-4.23050700	-0.28259900
		H	0.88167000	-4.87107800	-0.26022900
		H	2.37409100	-4.48783000	0.57245400
		H	2.31409700	-4.46490500	-1.18371300
		Se	0.03478000	1.74409000	-0.14630200
		C	-4.63612400	1.17924800	-0.01664300
		C	-3.95693400	1.24706200	-1.23469100
		H	-4.50910400	1.17216700	-2.15566500
		C	-2.58567700	1.41245700	-1.26657500
		H	-2.06909400	1.48391300	-2.20442800
		C	-1.85699600	1.50673100	-0.08862100
		C	-2.52999400	1.44879400	1.12368700
		H	-1.98194300	1.53303600	2.04503500
		C	-3.90200300	1.28519400	1.16539400
		H	-4.40852800	1.24012500	2.11386100
		N	-6.00681200	0.96145900	0.01832500
		H	-6.49755100	1.25469300	-0.79819200
		H	-6.46002700	1.28177600	0.84626000
		O	0.29001700	2.27561900	-2.20310100
		O	0.49804900	2.73022200	-4.14759400
		H	1.43875900	2.63380100	-4.12346800

		C	1.46996400	-2.70713700	-0.24639200
		C	1.28304700	-1.78980400	-1.27656700
		H	1.48106300	-2.08649700	-2.29270500
		C	0.85248600	-0.49774600	-1.02108800
		H	0.70990800	0.19894000	-1.82974700
		C	0.59086500	-0.10264000	0.28520500
		C	0.77755100	-1.00563400	1.32123400
		H	0.58409000	-0.70353700	2.33454000
		C	1.21431200	-2.29405400	1.05629300
		H	1.35682800	-2.98125800	1.87264000
		C	1.91647800	-4.12023000	-0.53917200
		H	1.06181100	-4.75840300	-0.74770300
**Products2'**<br><br>**n$_i$ = 0**	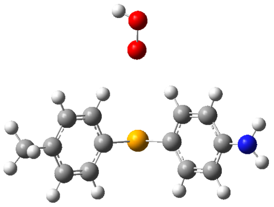	H	2.44699000	-4.54741700	0.30405500
		H	2.56887000	-4.15438200	-1.40438400
		Se	0.02101700	1.69003900	0.65848200
		C	-4.61606800	1.32333700	-0.10209000
		C	-3.73702400	1.54836900	-1.16292300
		H	-4.12684800	1.64526200	-2.16187500
		C	-2.37513500	1.65004700	-0.94511100
		H	-1.71158900	1.82058900	-1.77690700
		C	-1.85668500	1.51885600	0.33908500
		C	-2.72938700	1.29535400	1.39546500
		H	-2.34223900	1.19735500	2.39362800
		C	-4.09230200	1.19859000	1.18479100
		H	-4.75309800	1.02417100	2.01630300
		N	-5.97838200	1.17351700	-0.32998500
		H	-6.31429600	1.62179100	-1.15448500
		H	-6.55739500	1.38236700	0.45408300
		O	0.09465500	2.01424900	-3.35981800
		O	-0.13278200	1.84593300	-4.78942700
		H	0.73617200	1.69738000	-5.12546100

Table S18. Total electronic energies for the compounds involved in the restoration of the initial oxidation state of Se on diarylselenide at the HF/6-31G(d,p) level.

Compound[a]	EE[b]	Charge[c]
**Reactants'[d]**	-3026.994729	1
**Intermediate1'**	-3027.195147	1
**TS1'**	-3102.625559	0
**Products1'**	-3102.726341	0
**Intermediate2'**	-3026.734535	0
**TS2'**	-3102.132076	-1
**Products2'**	-3102.194738	-1

[a] Multiplicty = 1. [b] in Hartree. [c] in atomic units. [d] Single-point energy calculation conducted based on a hypothesized structure with OH^- being placed 4 Å away from Se.

Table S19. The electronic energy (in Hartree) of fully characterized stationary points on the PES for reaction step 1 with selected -R groups calculated at HF/6-31G(d,p) level of theory

-R	Reactants	TS	Products
**-NO_2**	-3379.284628	-3379.250485	-3379.348518
**-NH_2**	-3230.849765	-3230.816327	-3230.910647
**-SH**	-3573.321018	-3573.287599	-3573.386648
**-OH**	-3250.675137	-3250.641560	-3250.735330
**-H**	-3175.810160	-3175.776687	-3175.870073
**-CH_3**	-3214.850527	-3214.817167	-3214.910407

Table S20. A summary of E_a and ΔH (in kJ/mol) for the reaction step 1 with selected -R groups calculated at HF/6-31G(d,p) level of theory

-R	E_a	ΔH
**-NO_2**	89.64	-167.74
**-NH_2**	87.79	-159.85
**-SH**	87.74	-172.31
**-OH**	88.16	-158.04
**-H**	87.88	-157.30
**-CH_3**	87.59	-157.21

Table S21. The electronic energy (in Hartree) of additional fully characterized stationary points on the PES for reaction step 1 with selected -R groups calculated at HF/6-31G(d,p) level of theory

-R	Reactants	TS	Products
**-O^-**	-3250.171553	-3250.138411	-3250.237553
**-$C(CH_3)_3$**	-3331.957671	-3331.924241	-3332.018098
**-COOH**	-3363.441318	-3363.407757	-3363.500593
**-COO^-**	-3362.954762	-3362.921534	-3363.015334
**-CHO**	-3288.544121	-3288.510435	-3288.603252
**-CN**	-3267.548337	-3267.514541	-3267.607011

Table S22. A summary of E_a and ΔH (in kJ/mol) for the reaction step 1 with additional systems with selected -R groups calculated at HF/6-31G(d,p) level of theory

-R	E_a	ΔH
**-O^-**	87.01	-173.28
**-$C(CH_3)_3$**	87.77	-158.65
**-COOH**	88.11	-155.63
**-COO^-**	87.24	-159.03
**-CHO**	88.44	-155.25
**-CN**	88.73	-154.05

Table S23. Cartesian coordinates (in Angstrom, Å) of the TS in the Se oxidation reaction step 1 with -NO_2 as the -R group at the *para* position calculated at HF/6-311+G(d,p) level of theory.

**Compound & n_i**	**Structure**	**Atom coordinates**			
**TS** With -NO_2 as the -R group **n_i = 1**	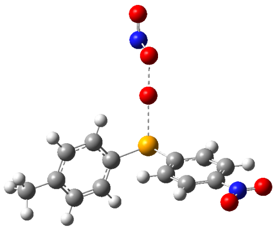	C	-3.31297400	-2.96712900	0.58850800
		C	-2.61325800	-3.19515900	-0.59426100
		H	-2.69255100	-4.14877300	-1.08625200
		C	-1.81671900	-2.21145600	-1.15267600
		H	-1.28998800	-2.40841200	-2.06848100
		C	-1.69611600	-0.97660200	-0.52623900
		C	-2.38997000	-0.73281700	0.64892100
		H	-2.30724700	0.22266400	1.13109200
		C	-3.18862900	-1.72477300	1.19874400
		H	-3.71797500	-1.52384800	2.11351100
		C	-4.20393400	-4.03615000	1.17346800
		H	-4.36635700	-3.87338300	2.23233300
		H	-3.77045300	-5.02082800	1.03916500
		H	-5.17344000	-4.03224800	0.68256600
		Se	-0.63380500	0.40733900	-1.32186000
		C	3.68240200	-0.38784100	0.25525900
		C	3.45899900	0.55963900	-0.72873700
		H	4.27129300	1.12658800	-1.13635200
		C	2.17005600	0.75584400	-1.17823200
		H	1.98989200	1.48494000	-1.94657300
		C	1.11817500	0.01630000	-0.64279100
		C	1.36453000	-0.92780100	0.34468400
		H	0.56545200	-1.50401000	0.76691300
		C	2.65464700	-1.13514900	0.79720900
		H	2.85210800	-1.86225100	1.55877100
		N	5.04284400	-0.60386700	0.73324100
		N	-2.46340500	4.48168500	0.54894700
		O	-2.77054900	5.48067800	1.15112900
		O	-1.50409300	3.85630500	0.99582600
		O	-1.13888900	2.32717400	-0.03462100
		O	5.91410200	0.06019000	0.26663600
		O	5.22059100	-1.43386300	1.56829200

Table S24. Cartesian coordinates (in Angstrom, Å) of the TS in the Se oxidation reaction step 1 with $-NH_2$ as the -R group at the *para* position calculated at HF/6-311+G(d,p) level of theory.

This structure is reported as Int 1 in
Table S3. Cartesian coordinates (in Angstrom, Å) of characterized stationary points on the Se oxidation reactions calculated at HF/6-311G+(d,p) level of theory.

Table S25. Cartesian coordinates (in Angstrom, Å) of TS1 in the Se oxidation reaction step 1 with -SH as the -R group at the *para* position calculated at HF/6-311+G(d,p) level of theory.

**Compound & n_i**	**Structure**	**Atom coordinates**			
**TS1** With -SH as the -R group **n_i = 1**	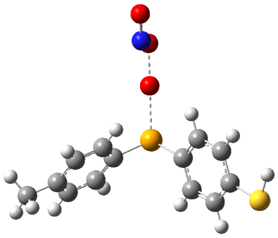	C	-3.37872400	-2.40278300	1.02393700
		C	-3.12436200	-2.71459900	-0.30266800
		H	-3.61070200	-3.55988600	-0.75697400
		C	-2.24180600	-1.95384700	-1.06224000
		H	-2.05672400	-2.22226800	-2.08629200
		C	-1.60478700	-0.86137400	-0.50079100
		C	-1.86101500	-0.52944000	0.82643400
		H	-1.38495900	0.32610200	1.26815400
		C	-2.73028100	-1.29703200	1.57646200
		H	-2.91502100	-1.03022900	2.60288000
		C	-4.33013500	-3.22649300	1.85845100
		Se	-0.42080900	0.21278100	-1.57324400
		C	3.73226400	-0.74296800	0.43158500
		C	3.11078700	0.47629200	0.65534500
		H	3.57006100	1.21854500	1.28203700
		C	1.88153800	0.74762400	0.07165100
		H	1.40273600	1.69132800	0.25055000
		C	1.26731900	-0.19266800	-0.73984600
		C	1.89848300	-1.40880700	-0.97266000
		H	1.44200200	-2.14450600	-1.60909400
		C	3.11942100	-1.68647500	-0.38676800
		H	3.59371500	-2.63329700	-0.57311800
		N	-1.25192800	4.21953100	1.04640800
		O	-1.46371700	5.30866100	1.51862000
		O	-1.19305300	4.16693500	-0.18061700
		O	-0.85360500	2.43555000	-0.78027900
		H	-5.13982400	-2.61176700	2.23966100
		H	-3.81771700	-3.65932400	2.71237200
		H	-4.76165200	-4.03327200	1.27832400
		S	5.30509000	-1.16337500	1.15042600

		H	5.51844000	-0.03245700	1.81497400

Table S26. Cartesian coordinates (in Angstrom, Å) of the TS1 in the Se oxidation reaction step 1 with -OH as the -R group at the *para* position calculated at HF/6-311+G(d,p) level of theory.

**Compound & n_i**	**Structure**	**Atom coordinates**		
**TS1** With -OH as the -R group **n_i = 1**	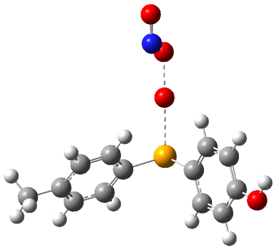	C	-3.71272600	-1.52019500	0.88352800
		C	-3.50623100	-1.85586700	-0.44520900
		H	-4.21040800	-2.49145800	-0.95283800
		C	-2.39358500	-1.39066900	-1.13880000
		H	-2.25273000	-1.67560000	-2.16553300
		C	-1.47310100	-0.57162400	-0.50945700
		C	-1.67497900	-0.21752200	0.82165500
		H	-0.97557600	0.42792900	1.32004200
		C	-2.77522800	-0.69441900	1.50643700
		H	-2.91385200	-0.41593400	2.53703400
		C	-4.91648600	-2.02070600	1.64551100
		Se	0.03105700	0.10660500	-1.50201300
		C	3.63596300	-1.78922300	0.84840300
		C	3.32872200	-0.44866400	1.01241900
		H	3.91727000	0.16549100	1.67141200
		C	2.25575700	0.10106100	0.32534700
		H	2.01762000	1.13951600	0.45525000
		C	1.49001300	-0.67833000	-0.52634100
		C	1.81642100	-2.02142200	-0.69310100
		H	1.24068200	-2.63814500	-1.35888600
		C	2.87691400	-2.57895400	-0.00751200
		H	3.12845400	-3.61629900	-0.13161500
		N	-0.01644300	4.33013800	0.89913900
		O	0.01767000	5.45967200	1.32011200
		O	0.18840700	4.19854700	-0.30604100
		O	0.12563800	2.40869900	-0.82017300
		H	-5.55193500	-1.19329600	1.94748900
		H	-4.61304600	-2.54589900	2.54587000
		H	-5.50856200	-2.69752200	1.04118400
		O	4.66243800	-2.38018900	1.49135500
		H	5.11929200	-1.76895800	2.04575500

Table S27. Cartesian coordinates (in Angstrom, Å) of the TS1 in the Se oxidation reaction step 1 with -H as the -R group at the *para* position calculated at HF/6-311+G(d,p) level of theory.

**Compound & n_i**	**Structure**	**Atom coordinates**			
**TS1** With -H as the -R group $n_i = 1$	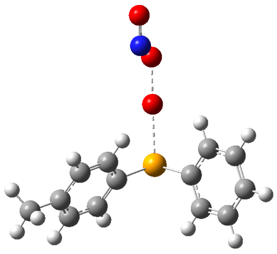	C	-3.76814600	1.05598200	0.67326400
		C	-3.75561100	0.48986900	-0.59193100
		H	-4.67775800	0.36335300	-1.13145900
		C	-2.56614500	0.07224500	-1.17971600
		H	-2.58644200	-0.37225600	-2.15797200
		C	-1.36660500	0.22426200	-0.50697100
		C	-1.36371100	0.80343500	0.75893600
		H	-0.43881600	0.94026200	1.28805900
		C	-2.55070700	1.20428700	1.33982200
		H	-2.53222000	1.64576000	2.32144200
		C	-5.05486400	1.50516800	1.32336300
		Se	0.27583100	-0.31551000	-1.35388200
		C	1.60950300	-3.86007800	1.43520800
		C	2.27866500	-2.64854500	1.48087400
		H	3.11091100	-2.51580900	2.14876700
		C	1.87611700	-1.59696600	0.66742000
		H	2.38886400	-0.65490700	0.70286500
		C	0.80296900	-1.76425000	-0.19511500
		C	0.13745800	-2.98388400	-0.25034500
		H	-0.68694500	-3.12035600	-0.92617400
		C	0.53697500	-4.02638300	0.56928300
		H	0.01757100	-4.96693600	0.52698800
		N	2.90493100	3.22201600	0.70279100
		O	3.67585400	4.08387900	1.04514500
		O	3.10116200	2.73440400	-0.40861300
		O	1.85078400	1.40801600	-0.80597700
		H	-5.01692800	2.56350700	1.56274000
		H	-5.22696000	0.96590500	2.25000100
		H	-5.90300100	1.33574700	0.67098800
		H	1.92115300	-4.67227900	2.06723600

Table S28. Cartesian coordinates (in Angstrom, Å) of the TS1 in the Se oxidation reaction step 1 with -CH_3 as the -R group at the *para* position calculated at HF/6-311+G(d,p) level of theory.

**Compound & n$_i$**	**Structure**	**Atom coordinates**			
**TS1** With -CH_3 as the -R group **n$_i$ = 1**	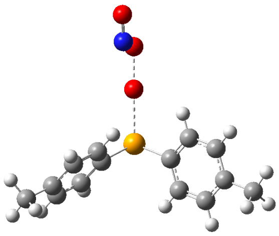	C	-3.49799000	-1.96674400	0.85831900
		C	-3.26056200	-2.23669500	-0.48021200
		H	-3.88609400	-2.93762800	-1.00453500
		C	-2.21645000	-1.62070200	-1.16267300
		H	-2.04844000	-1.85646100	-2.19771100
		C	-1.39802900	-0.71493600	-0.51160500
		C	-1.63323100	-0.42582900	0.82971800
		H	-1.01384100	0.28517200	1.34456400
		C	-2.66387100	-1.05147400	1.50283000
		H	-2.82957600	-0.82096900	2.54121900
		C	-4.62617800	-2.63280300	1.60903200
		Se	0.00830300	0.16914300	-1.48416400
		C	3.83865400	-1.40969200	0.79249600
		C	3.41922800	-0.09474400	0.92418400
		H	3.97539400	0.58398600	1.54685100
		C	2.28385800	0.36792000	0.26726400
		H	1.97122300	1.38786200	0.38591100
		C	1.55380500	-0.48640900	-0.53969800
		C	1.97135700	-1.80580200	-0.69224300
		H	1.42138500	-2.47768100	-1.32604000
		C	3.09460800	-2.25880400	-0.02791000
		H	3.40254900	-3.28268100	-0.15315400
		N	-0.71073800	4.33634100	0.91496300
		O	-0.80627100	5.45767800	1.34845400
		O	-0.21860100	4.23291700	-0.20692000
		O	-0.13104000	2.45225300	-0.75255300
		H	-5.33491600	-1.89499600	1.97306300
		H	-4.24908500	-3.17684800	2.46958700
		H	-5.16020900	-3.33011700	0.97480400
		C	5.06262200	-1.92199100	1.51333200
		H	5.57713200	-1.12026700	2.02930300
		H	4.78958400	-2.67499700	2.24690200
		H	5.75812200	-2.38016900	0.81722800
